# CD44-Mediated Poor Prognosis in Glioma Is Associated With M2-Polarization of Tumor-Associated Macrophages and Immunosuppression

**DOI:** 10.3389/fsurg.2021.775194

**Published:** 2022-02-03

**Authors:** Yong Xiao, Kun Yang, Zhen Wang, Mengjie Zhao, Yanxiang Deng, Wei Ji, Yuanjie Zou, Chunfa Qian, Yong Liu, Hong Xiao, Hongyi Liu

**Affiliations:** ^1^Department of Neurosurgery, Nanjing Brain Hospital Affiliated to Nanjing Medical University, Nanjing, China; ^2^Department of Neuro-Psychiatric Institute, Nanjing Brain Hospital Affiliated to Nanjing Medical University, Nanjing, China; ^3^Department of Biomedical Engineering, Yale University, New Haven, CT, United States; ^4^Department of Neurosurgery, Wuxi People's Hospital of Nanjing Medical University, Wuxi, China

**Keywords:** *CD44*, glioma, M2, tumor-associated macrophage, spatial, single cell, immunosuppression

## Abstract

**Background:**

Glioma is the most common primary brain tumor with a poor prognosis. Key genes that are negatively related to prognosis may provide the therapy targets to cure glioma. To clarify the role of *CD44* in glioma, we explored its function at bulk-transcriptome, spatial and single-cell transcriptome levels.

**Methods:**

In total, expression profiles with survival data of whole-grade glioma from The Cancer Genome Atlas (TCGA) and the Chinese Glioma Genome Atlas (CGGA), RNA-seq data with anatomic information of glioblastoma (GBM) from the Ivy Glioblastoma Atlas Project, RNA-sequencing (RNA-seq) data from recurrent GBM receiving adjuvant anti*-PD-1* immunotherapy accessed through GSE121810, and single-cell RNA-seq data of GBM under accession GSE103224 were enrolled in this study. *CD44*-specific findings were further analyzed by R language.

**Results:**

*CD44* is positively correlated with WHO grade of malignancy and is negatively related to prognosis in glioma. Meanwhile, *CD44* predominantly expresses in GBM mesenchymal subtype, and gene ontology (GO) and Kyoto Encyclopedia of Genes and Genomes (KEGG) analyses reveal that *CD44* positively coexpressed genes are closely related to glioma immunity. Moreover, *CD44*+ cells mainly distribute in perinecrotic region with high expression of immune factors. At single-cell resolution, only malignant tumor cells, tumor-associated macrophages (TAMs), and T cells express *CD44* in GBM. *CD44*+ malignant tumor cells are in mesenchymal-1-like (MES1-like) cellular state, and *CD44*+ TAMs are in M2 phenotype. *CD44*+ T cells have high expression of both *PD-1* and *PD-L1*. *CD44* and its directly interacted inhibitory immunomodulators are upregulated in patients with nonresponder recurrent GBM treated with *PD-1* blockade therapy.

**Conclusion:**

Our work demonstrates that *CD44*, a new M2 TAM biomarker, is involved in immune suppressor and promote glioma progression in glioma microenvironment. These results expand our understanding of *CD44*-specific clinical and immune features in glioma.

## Introduction

The most common primary brain tumor in adults is glioma. Most of the patients with glioma confront recurrence and death even though undergoing current standard therapy. The 5-year survival rate varies from 50 to 81% in WHO II glioma and ranges from 30 to 57% in WHO III glioma, but is only 5.5% in glioblastoma (GBM, WHO IV) (1). Despite improvements in standard therapy, patients with the most aggressive type, GBM, have a median survival time of only 15 months (2). In the past decades, many new antineoplastic treatments have achieved good results in various tumors except for glioma. Bevacizumab, the only approved small molecule drug for glioma, also failed to prolong the overall survival (OS) of newly diagnosed patients with GBM (3). Although the chimeric antigen receptor (CAR)-T cell therapy succeeds in some malignancies, it does not work well in patients with glioma to improve OS and is still at an early clinical investigation stage (4). Given these clinical challenges of glioma, there is a considerable interest in exploring the molecular mechanisms related to the short survival of patients with glioma, which may be the new therapy targets for glioma.

*CD44* molecule is a complex transmembrane adhesion glycoprotein and participates in a wide variety of cellular functions including cell adhesion, migration, proliferation, apoptosis, and angiogenesis, which, when pathologic, are the characteristics of malignancy (5). Unsurprisingly, *CD44* is aberrantly upregulated among diverse tumors, including pancreatic cancer, breast cancer, prostate cancer, head and neck squamous cell carcinoma, and gastrointestinal cancer (6). *CD44* is a well-known marker of glioma cancer stem cells (CSCs) and plays important roles in tumor initiation and progression (7). In addition, WHO grades II and III patients with glioma with high *CD44* mRNA expression faced poor survival compared to low *CD44* mRNA level in an independent manner (8). *CD44* expression level was elevated after irradiation or temozolomide treatment in mouse glioma model (9), which indicated *CD44* is involved in resistance to chemoradiation in glioma. Therefore, *CD44* could be the candidate for glioma therapy.

However, the precise mechanisms underlying *CD44*-mediated glioma initiation and progression have not been completely elucidated. *CD44* also plays important roles in diverse physiological processes, such as organ development, hematopoiesis, and diverse immune functions including T cell and lymphocyte activation (10). But we know few about the immune functions of *CD44* in tumor until now. A deeper understanding of the role of *CD44* in glioma will guide promising research in novel glioma therapeutic strategy. Herein, we integrated bulk RNA-sequencing data (RNA-seq) and single-cell RNA-seq data to investigate the immunosuppressive role of *CD44* in glioma. Additionally, we further validated our findings with immunohistochemistry (IHC) and immunofluorescence (IF) staining data.

## Methods

### Bulk RNA-Seq Data

RNA expression data for human gliomas were downloaded from The Cancer Genome Atlas (TCGA) (11) and the Chinese Glioma Genome Atlas (CGGA) (12). These datasets contain bulk mRNA expression profiles and corresponding clinical information of patients with glioma. A total of 702 glioma samples from TCGA and 693 glioma samples from CGGA were used in our study. At present, the WHO's classification on glioma utilized by TCGA and CGGA is the fourth WHO's classification of tumors of the central nervous system (13). RNA-seq data of 122 GBM samples with anatomic information from the Ivy Glioblastoma Atlas Project (IGAP) (14) were used in this study. RNA-seq data from 15 patients with recurrent GBM treated with adjuvant anti*-PD-1* immunotherapy were accessed through GSE121810.

### Survival Analysis

Survival curves were performed by Kaplan–Meier analysis between *CD44* higher and lower group and were tested for significance using the Mantel–Cox log-rank test. Hazard ratio (HR) and confidence interval (CI) were also computed to confirm the prognostic value of *CD44* in patients with glioma. Furthermore, Cox proportional hazards model, including patients' age and gender, was used to evaluate the predictor effect of *CD44*. A value of *p* < 0.05 was considered statistically significant.

### Immune Infiltration Analysis

Estimation of infiltrating stromal cells and immune cells in glioma samples was accomplished by the method ESTIMATE (15). Then, the xCell pipeline (16) was adopted to explore the relationship between *CD44* expression level and glioma-infiltrating cell types. Because some cell types from xCell reference database do not distribute in the brain and glioma, we only used 41 cell states listed in Supplementary Figure 4 in this part analysis. Furthermore, we utilized CIBERSORTx portal (17) to infer immune cell compositions among TCGA and CGGA glioma using a set of 22 human immune cell reference profiles and predicting their absolute composition ratio within individual glioma sample.

### Weighted Gene Coexpression Network Analysis

Weighted gene coexpression network analysis (WGCNA), a bioinformatics algorithm (18), was used to generate unsigned coexpression networks in TCGA and CGGA glioma. WGCNA applies topological overlap measure, a robust measure of network interconnectedness and measures the connection strength between two adjacent transcripts and all other transcripts in a network, to cluster genes into network modules. The minimum size of modules was 30 transcripts and were randomly color labeled. Next, we related the gene modules to clinical traits and selected the module of interest which contained the *CD44* and had the highest correlation coefficient with *CD44* expression level. Intramodular connectivity of transcripts was used to identify hub genes in the module of interest.

### Gene Set Enrichment Analysis

Gene ontology (GO) (19) and Kyoto Encyclopedia of Genes and Genomes (KEGG) (20) analyses were used to explore the biological function of gene sets of interest. Pearson's correlation coefficients between *CD44* and all other genes were calculated, and *CD44*-associated genes were defined as genes with |r| >0.5 in TCGA glioma dataset, |r| > 0.25 in CGGA glioma dataset, and |r| > 0.5 in IGAP GBM dataset. Additionally, GSEA (Linux_4.1.0) software was used to determine whether the gene set of *PD-1* signaling (http://www.gsea-msigdb.org/gsea/msigdb/cards/REACTOME_PD_1_SIGNALING.html) shows statistically significant difference between anti-*PD-1* immunotherapy responder and nonresponder subgroups.

### Protein–Protein Interaction Network

The immunomodulator genes were downloaded from the study introduced by Thorsson (21). These immunomodulators with *CD44* were imported into the STRING website (https://string-db.org/) to construct protein–protein interaction network. All the parameters were default.

### Differentially Expressed Gene Analysis

Raw count matrices of 15 patients with recurrent GBM treated with adjuvant anti-*PD-1* immunotherapy were utilized in this part analysis. These 15 patients were divided into responder and nonresponder subgroups based on OS cutoff for 300 days. The patient with OS greater than 300 days was labeled as therapy responder, but the one with OS lower than 300 days was regarded as nonresponder. In addition, differentially expressed genes (DEGs) between nonresponder and responder groups were identified by the package DESeq2 (1.32.0) (22).

### Data Processing in Single Cell

The human GBM signal cell RNA-seq data were downloaded from The Gene Expression Omnibus (GEO) (23) under accession number GSE103224. The expression matrices were analyzed using the R package Seurat (24). We removed all cells from the downstream analysis where >10% transcripts aligned to the mitochondrial genome, or which had either fewer than 400 or more than 2,500 RNA counts. Besides, gene was filtered out when it expressed in less than 10 cells among all the 7 GBM samples. The remaining 28,825 genes in 17,357 cells passed quality control and were used into further analysis. Malignant tumor cells were distinguished from stromal cells by copy number variants (CNVs), and nontumor cells were identified using known cell-type marker genes. FindMarkers in Seurat package was used to identify the DEGs between *CD44*+ and *CD44*- cell cluster. Additionally, the top 100 DEGs according to the avg_logFC were underwent the gene set enrichment analysis. Pairwise Pearson's correlation with *CD44* was established in *CD44*+ cell cluster, and the top 100 correlated genes according to correlation coefficient were used in subsequent enrichment analysis. We took the standard workflow of pySCENIC (0.11.2) (25) to build the regulon network of different cell types. Regulons were ranked by the regulon specificity score from high to low, and the top 20 regulons of each cell type were used to plot the heatmap among different cell types. The regulon specificity scores were scaled by cell types. The Monocle 2 package (2.14.0) (26) was applied to construct single-cell pseudotime trajectories. Considering the small number of oligodendrocytes, endothelial cells, pericytes, and T cells, only malignant tumor cells and TAMs were used in trajectory analysis, respectively. The DEGs were identified by Monocle 2, and these genes filtered by qval <0.05 were further used in pseudotime order analysis.

### Immunohistochemistry Staining

The IHC data of unique GBM subtype samples from the IGAP and of normal brain tissues and glioma samples from the Human Protein Atlas (https://www.proteinatlas.org/) were downloaded and used in this study.

### Immunofluorescence Staining

Formalin-fixed paraffin-embedded sections of glioma were collected and their pathological results were confirmed as glioma according to the 2016 WHO's classification of central nervous system tumors (13). Tumor sample use was approved by the Institutional Review Board at the Nanjing Brain Hospital Affiliated to Nanjing Medical University. Tumor sample was incubated with the first primary antibody against *CD44* (1:5000 for IF, Servicebio) overnight at 4°C and then with the first matching secondary antibody at room temperature for 50 min in dark condition. Then, it was incubated with the second antibody *CD163* (1:5000 for IF, Servicebio) overnight at 4°C and later with the second matching secondary antibody at room temperature for 50 min in dark condition. Slide was counterstained with DAPI for nuclei visualization. Finally, sample slide was imaged by Imaging System from Nikon. The CaseViewer software (3DHISTECH) can unmix and remove autofluorescence and analyze the multispectral images.

## Results

### *CD44* Expression Level Is Correlated With Glioma Grade, IDH Type, 1p19q State, and Recurrent Status

**Figure 1:**
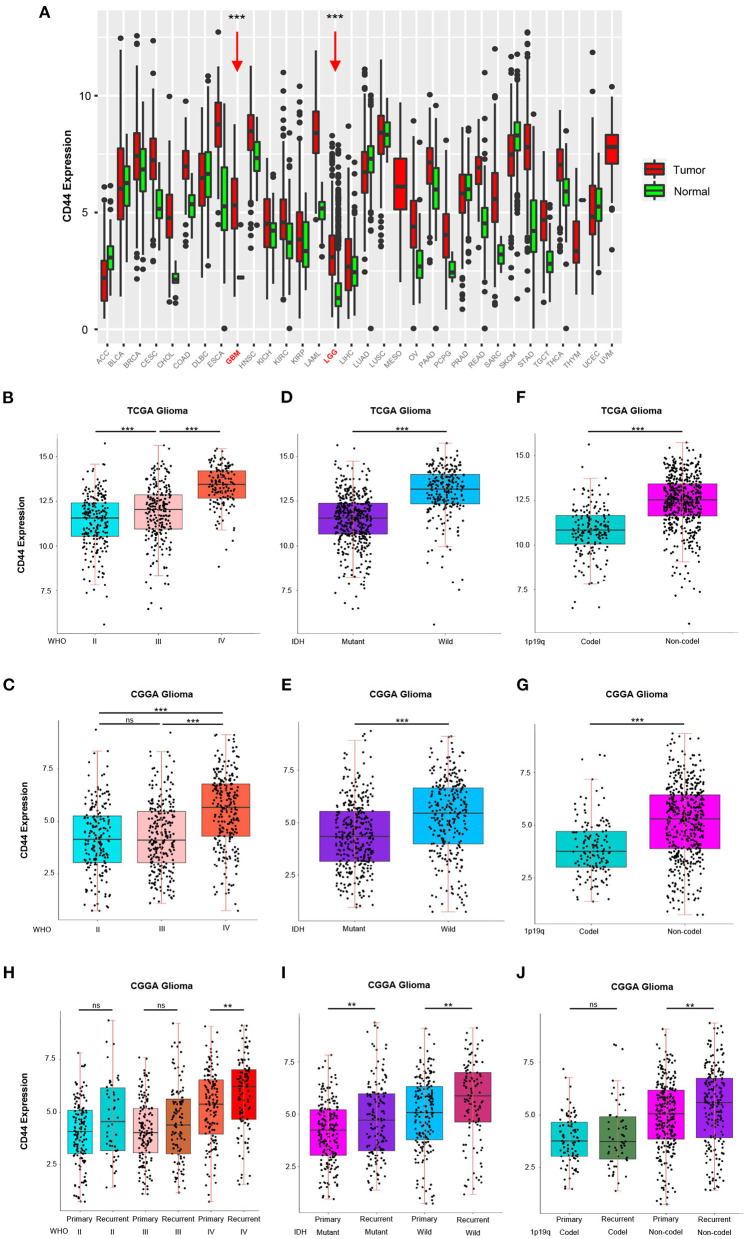
*CD44* is highly expressed in WHO grade IV, wild-type IDH, 1p19q non-codeletion and recurrent glioma. **(A)** The mRNA level of *CD44* is higher in GBM and LGG than normal brain. GBM and LGG are colored in red and pointed out by red arrows. *CD44* significantly increases in WHO grade IV form TCGA **(B)** and CGGA **(C)**. *CD44* significantly increases in wild-type IDH glioma form TCGA **(D)** and CGGA **(E)**. *CD44* significantly increases in 1p19q non-codeletion glioma form TCGA **(F)** and CGGA **(G)**. *CD44* significantly increases in recurrent glioma **(H–J)**. Tested by *t*-test: ***p < 0.001; **p < 0.01; ns, *p* ≥ 0.05.

To answer the question whether *CD44* is involved in malignant progress of glioma, we compared its expression level in different WHO grades, IDH types, 1p19q states, and recurrent status in TCGA and CGGA glioma mRNA-seq datasets. First, *CD44* expression in both low-grade glioma (LGG) and glioblastoma (GBM) is higher than normal brain tissues, which means *CD44* plays a role in glioma tumorigenesis (Figure 1A). It turns out that *CD44* is significantly upregulated in GBM (WHO IV) than that in WHO grades II and III gliomas in TCGA glioma dataset (Figure 1B). Additionally, CGGA RNA-seq data validate well to this result (Figure 1C). Additionally, the IHC data of normal brain tissues and glioma samples reveal that high-grade glioma has the highest *CD44* protein expression level, but that the normal brain tissue has the lowest *CD44* expression (Supplementary Figure 2). These shows that a higher *CD44* expression is associated with a higher malignancy in glioma.

Because *IDH1* mutation frequency is >68% in LGG, but only 12% in GBM (27), and 1p19q codeletion is more common in LGG than GBM (28), we studied *CD44* expression level in different IDH types and 1p19q states. In both the TCGA glioma and CGGA glioma, samples with strong *CD44* transcription are primarily having wild-type IDH, but the ones with low *CD44* expression have IDH mutation (Figures 1D,E), which can also be discovered among GBM groups (Supplementary Figures 3A,C). We also found a significant correlation between low *CD44* transcription level and 1p19q codeletion status in both TCGA and CGGA glioma (Figures 1F,G, Supplementary Figures 3B,D). Glioma patients with *IDH1* mutation have median OS three times longer than those without (27), codeletion of 1p and 19q is associated with better survival rates in glioma (29), and thus, low *CD44* expression patients with glioma may have better clinical outcomes.

Furthermore, we uncovered up expression of *CD44* after glioma recurrence in GBM, wild-type IDH, IDH mutation, and 1p19q non-codeletion state in CGGA glioma dataset (Figures 1H–J). Although multimodal treatments can prolong life, recurrence is inevitable in glioma, especially GBM, and it can obtain more malignant phenotype after recurrence (30). Herein, malignant glioma has relative higher *CD44* expression level, and *CD44* is involved in malignant progress of glioma.

### Transcriptional Level of *CD44* Effectively Predicts Survival of Patients With Glioma

**Figure 2:**
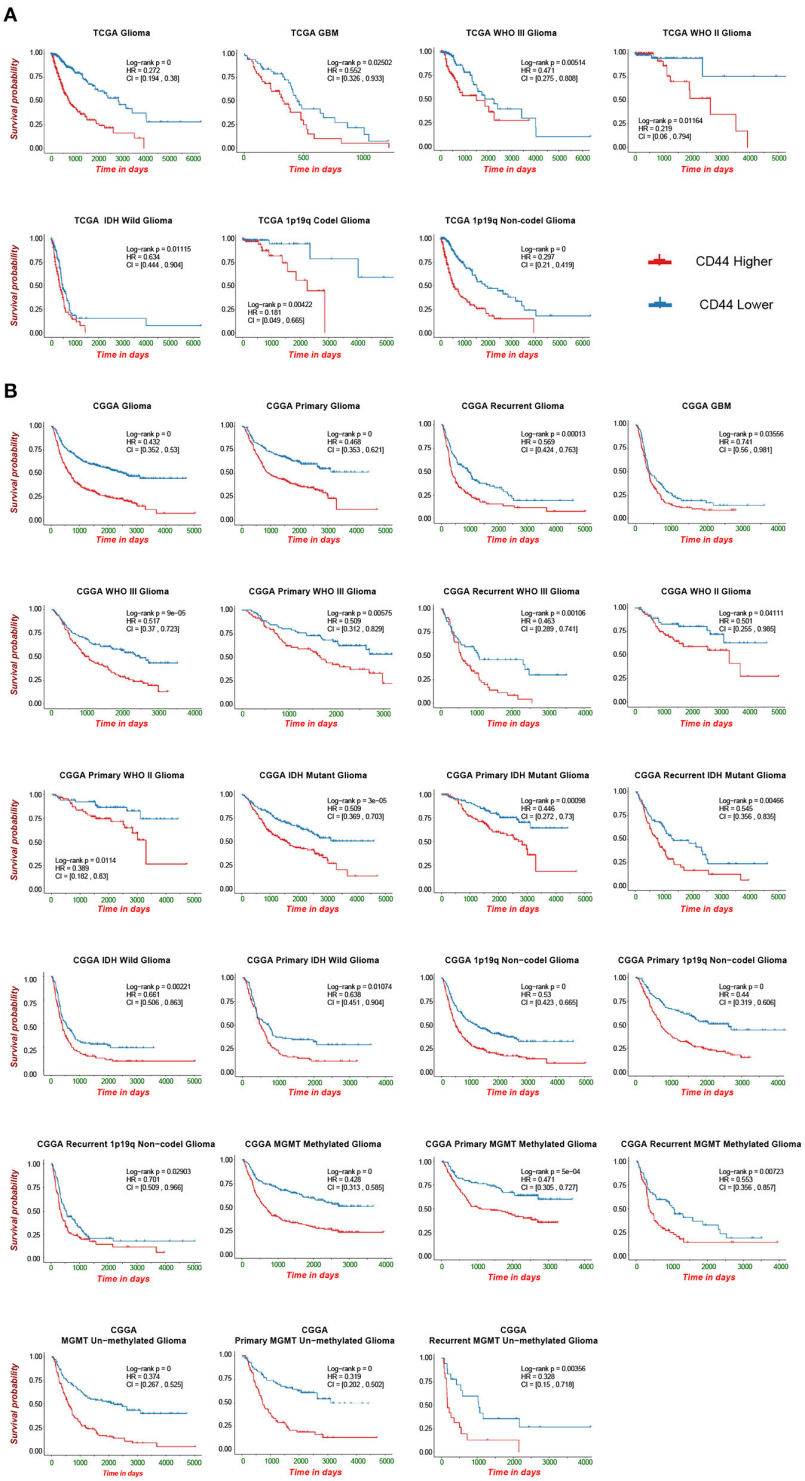
Survival analysis of *CD44* in patients with glioma. Survival analysis of *CD44* mRNA expression in glioma form TCGA **(A)** and CGGA **(B)**.

*CD44* transcription level is sufficient to predict OS of patients with glioma in TCGA and CGGA datasets (Figure 2). Compared with the lower expression of *CD44* group in whole-grade glioma, patients with higher *CD44* expression have significantly shorter survival. Given the obvious heterogeneity in glioma (31, 32), we further investigated the prognostic value of *CD44* among different WHO grades, IDH types, 1p19q states, and recurrent status. Interestingly, we observed the same prognostic tendency of *CD44* in these different compared groups (Figure 2). In addition, we did Cox-regression analysis of *CD44* with patients' age and gender, namely multivariate Cox-regression analysis, in different glioma groups to evaluate their predictor values in glioma. In the Cox proportional hazards model, patients with higher *CD44* expression level have higher mortality than those with lower *CD44* expression in different compared groups (HR>1, *p* < 0.05), except in 1p19q codeletion status (*p* > 0.05) (Table 1). These findings suggest that *CD44* could be a negative prognosticator in glioma.

**Table 1:** Multi-variate Cox-regression analysis of CD44 expression with Age and Gender.

**Dataset**	**Group**	**HR**	**95%CI**	***p***	
TCGA	All	1.5361	1.3669–1.726	5.68e-13	^***^
	WHO II	1.515	1.0482–2.189	0.0271	^*^
	WHO III	1.3258	1.0868–1.617	0.00543	^**^
	WHO IV	1.1447	0.9519–1.377	0.151	
	IDH mutant	1.2393	0.9928–1.547	0.057922	
	IDH wildtype	1.2441	1.0719–1.444	0.00406	^**^
	1p19q codeletion	1.487	0.9152–2.416	0.109	
	1p19q Non-codeletion	1.3618	1.1942–1.553	4.03e-06	^***^
CGGA	All	1.2832	1.2107–1.360	0	^***^
	WHO II	1.191	1.0026–1.415	0.0467	^*^
	WHO III	1.2822	1.1580–1.420	1.73e-06	^***^
	WHO IV	1.1233	1.0368–1.217	0.00448	^**^
	IDH mutant	1.3049	1.1815–1.441	1.52e-07	^***^
	IDH wildtype	1.1274	1.0453–1.216	0.00188	^**^
	1p19q codeletion	1.26	0.9945–1.596	0.0556	
	1p19q Non-codeletion	1.2018	1.1230–1.286	1.07e-07	^***^
	MGMT methylation	1.236	1.1297–1.352	3.78e-06	^***^
	MGMT Un-methylation	1.374	1.2460–1.514	1.79e-10	^***^
	Primary tumor	1.329	1.2164–1.451	2.69e-10	^***^
	Recurrent tumor	1.1573	1.0720–1.249	0.000184	^***^

*HR, hazard ratio; CI, confidence interval*;

*** *p < 0.001*,

** *p < 0.01*,

* *p < 0.05*.

### *CD44* Expression in Different Glioma Subtypes

**Figure 3:**
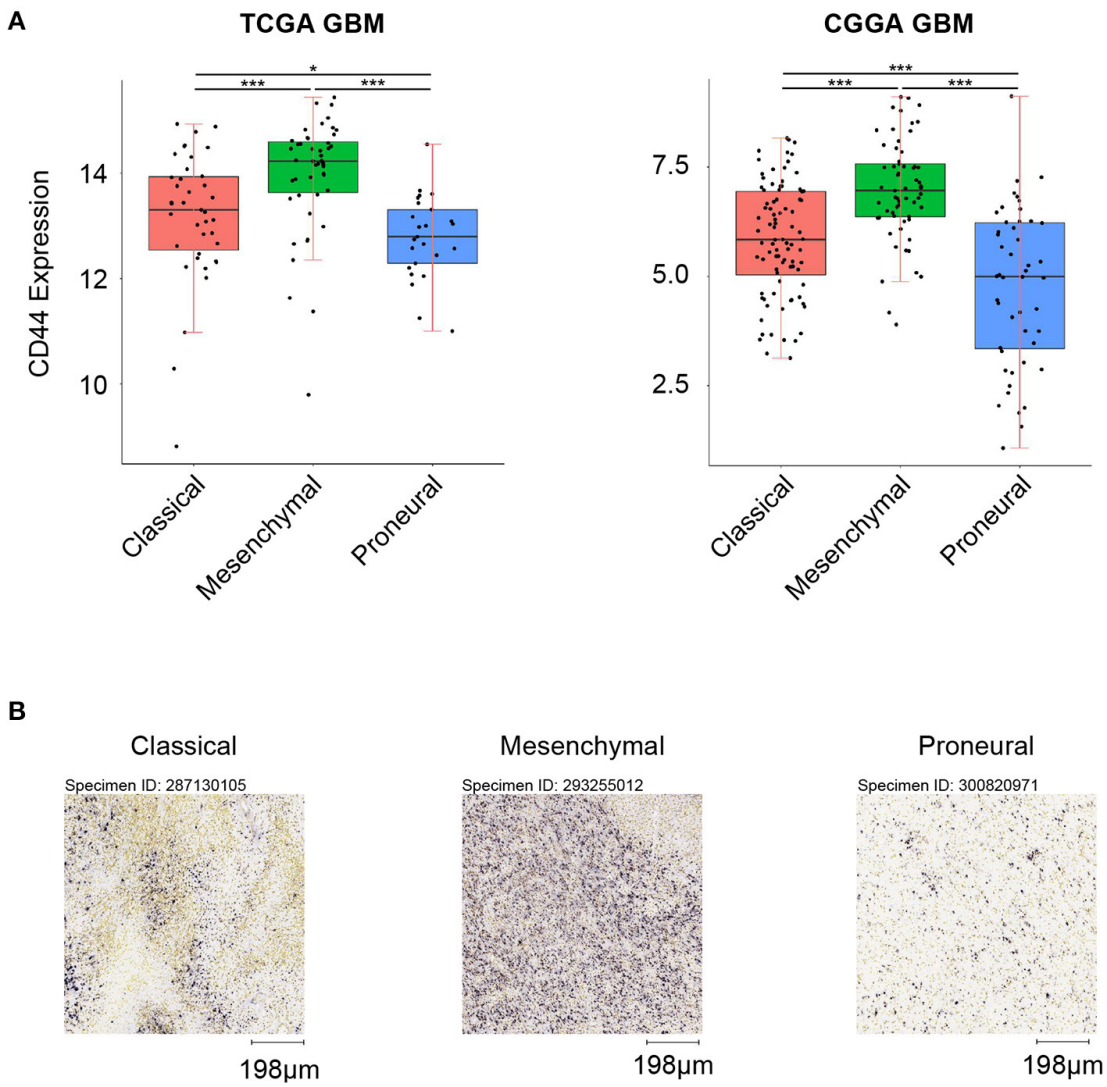
*CD44* expression in GBM subtypes. **(A)** *CD44* is highly upregulated in mesenchymal subtype in TCGA and CGGA GBM samples. **(B)** *CD44* protein expression is detected in three GBM subtypes, and mesenchymal subtype glioma has the highest *CD44* protein expression level. Scale, 198 μm. Tested by *t*-test: ***p < 0.001; *p < 0.05.

**Figure 4:**
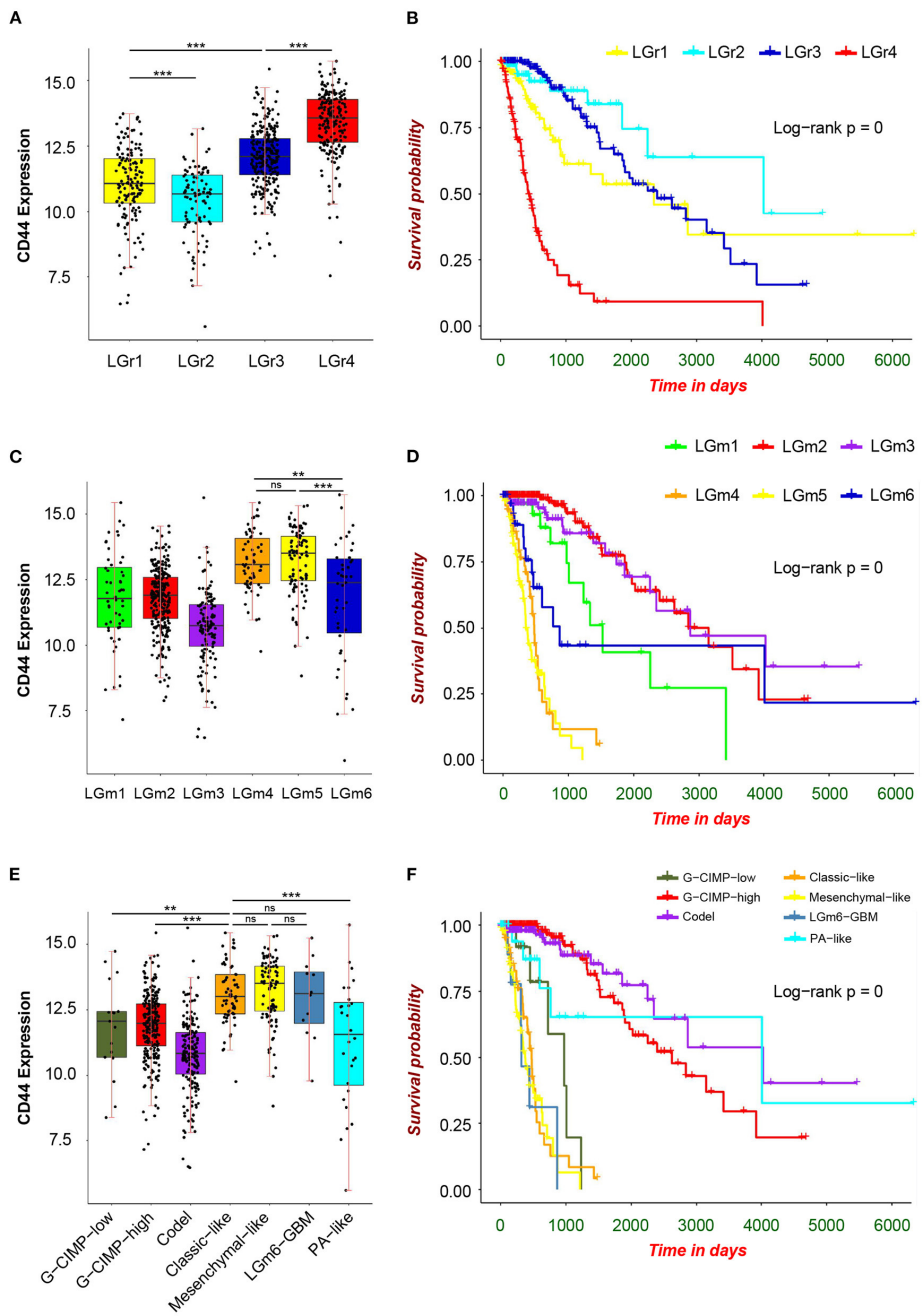
*CD44* expression in pan-glioma subtypes. Expression of *CD44* is elevated in LGr4 among pan-glioma transcriptome subtypes **(A)**, in LGm4 and LGm5 among pan-glioma DNA methylation subtypes **(C)** and in classic–like, mesenchymal–like, and LGm6–GBM among supervised pan-glioma subtypes **(E)**. These distinct glioma subtypes with high *CD44* expression are in bad clinical outcomes **(B,D,F)**. Tested by *t*-test: ***p < 0.001; **p < 0.01; ns, *p* ≥ 0.05.

First, we analyzed the distribution of *CD44* in different GBM transcription subtypes to obtain an overview of the molecular expression pattern of *CD44*. The *CD44* mRNA expression is quite different in the three different GBM characteristic subtypes (Figure 3A). Samples with high *CD44* expression are mainly concentrated in the mesenchymal subtype, which is verified by IHC results (Figure 3B). Considering the mesenchymal GBM subtype is featured with more infiltrating tumor-associated macrophages (TAMs) and immunosuppression (33), we speculated that *CD44* may play an important role in glioma immune environment. Next, we adopted the other glioma subtype model that portrays adult diffuse grades II, III, and IV gliomas comprehensively (34). They reveal that a subtype of IDH mutant glioma is associated with DNA hypermethylation and better outcome, and that a group of wild-type IDH diffuse glioma shows molecular similarity to pilocytic astrocytoma and relatively favorable survival. We explored the expression difference of *CD44* among pan-glioma transcriptome subtype (Figure 4A), pan-glioma DNA methylation subtype (Figure 4C), and supervised pan-glioma subtype (Figure 4E). Through comparing the survival in different subtypes (Figures 4B,D,F), we discovered that the subtypes with high *CD44* expression are related to bad clinical outcomes as well.

### *CD44* Correlates With Immune-Related Biological Response in Glioma

**Figure 5:**
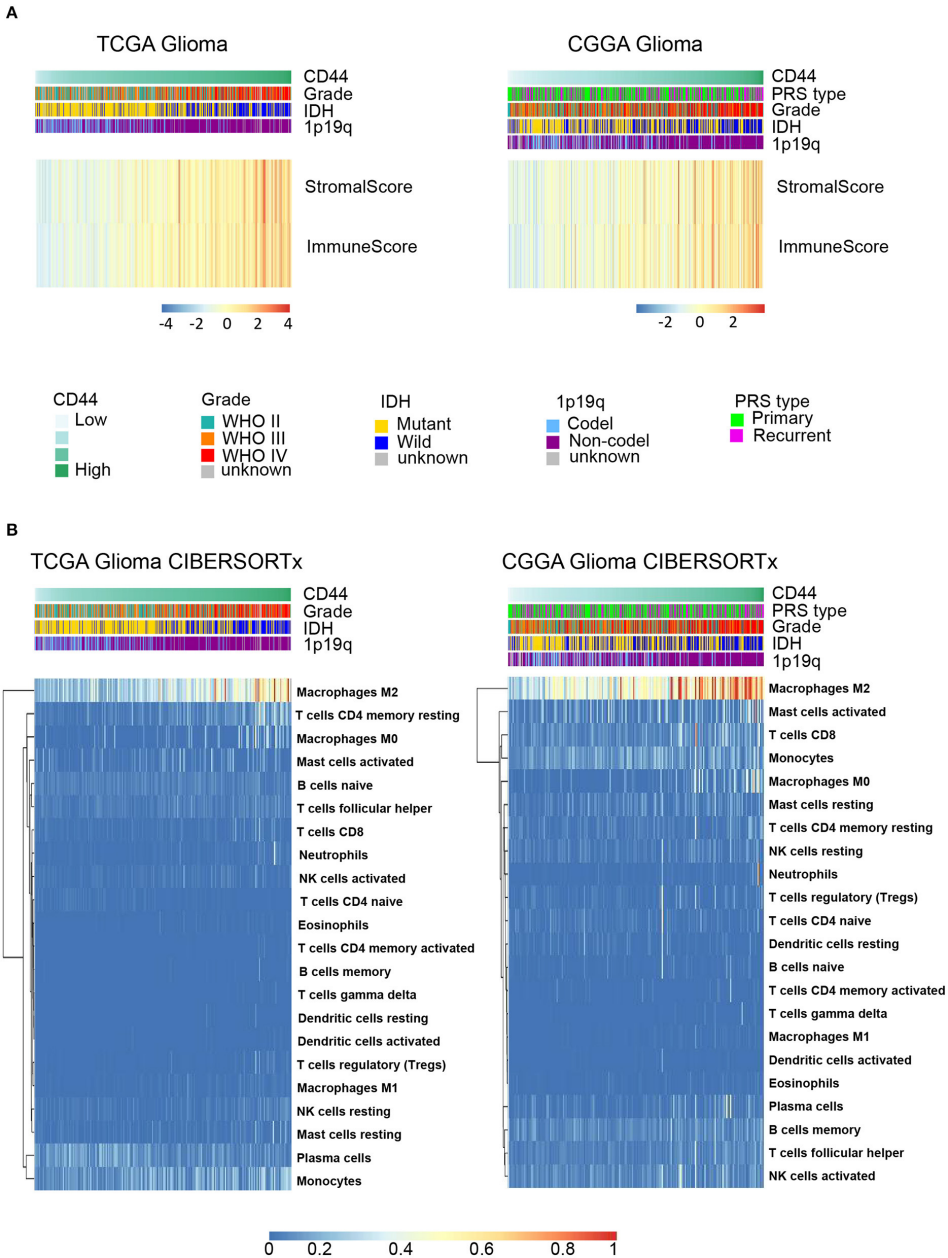
*CD44* is closely correlated with immune microenvironment in glioma. **(A)** Heatmap of immune score and stromal score in TCGA and CGGA gliomas. The higher *CD44* expression level the glioma has, the higher immune score and stromal score it is, which reflects glioma with elevated *CD44* expression has more infiltrating immune cells. **(B)** Component types of 22 human immune cells infiltrated into glioma were analyzed by CIBERSORTx in TCGA and CGGA glioma datasets. Glioma with high *CD44* expression has more infiltrating M2-type macrophages.

Because immune cells are the main participants in immune response, we characterized the relationship between *CD44* and the infiltrated immune cells in glioma. The results indicate that *CD44* is positively associated with the immune score and stromal score in TCGA and CGGA glioma databases (Figure 5A), which suggests that *CD44* does work in glioma immunity. Additionally, the standard CIBERSORTx pipeline was applied to further evaluate the relationship between *CD44* and infiltration of 22 immune cells in glioma microenvironment (17). In addition, the results show that *CD44* has the most significantly positive correlation with M2-type macrophages (Figure 5B). The analysis results generated by xCell workflow also confirmed the positive correlation between *CD44* expression level and macrophages M2 score (Supplementary Figure 4). It is well known that M2 TAMs accelerate tumor cell invasion and angiogenesis and suppress antitumor immunity (35), which explains why *CD44* can be prognostic biomarker and confirms immunity function of *CD44*.

**Figure 6:**
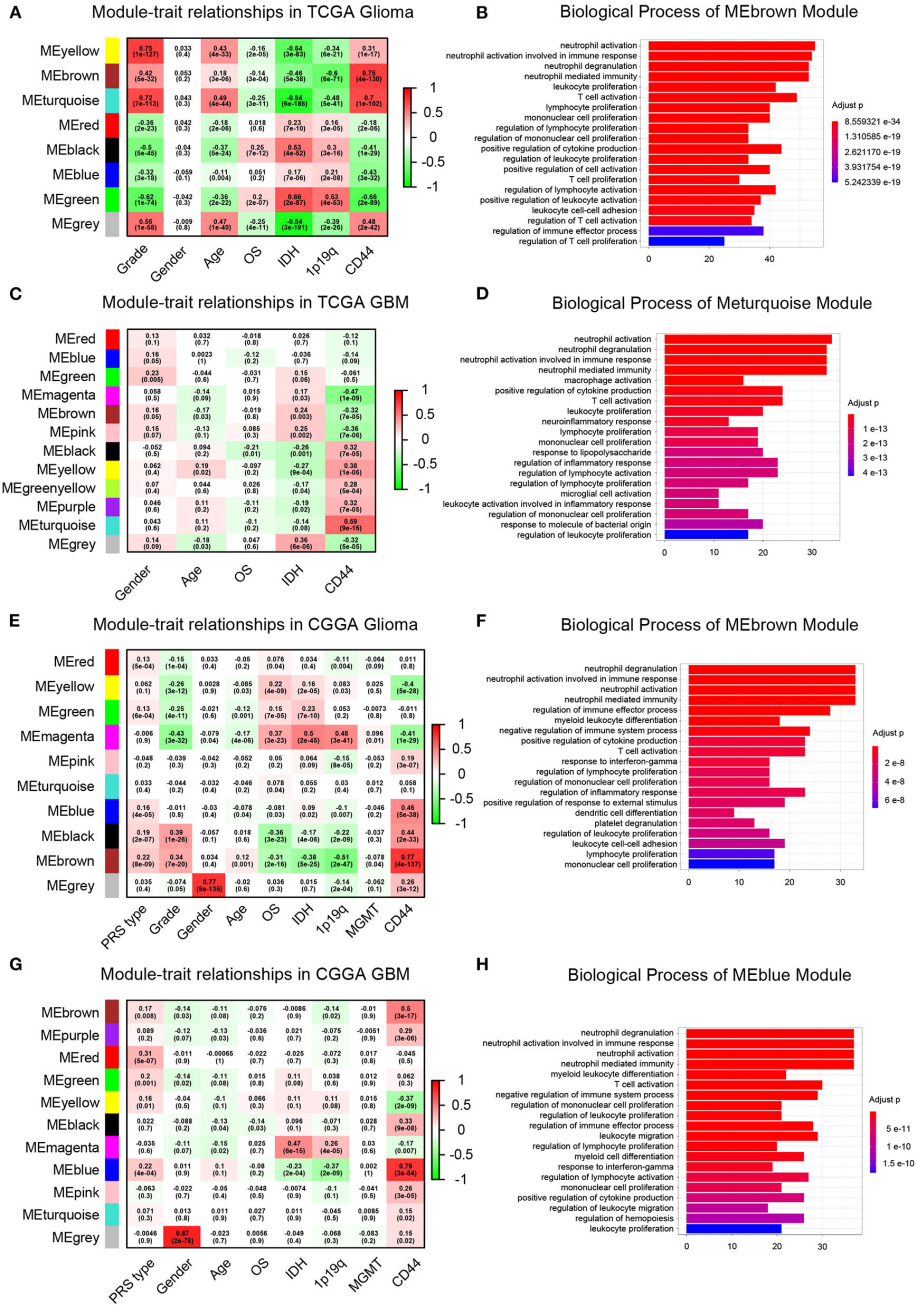
WGCNA analysis and enrichment analysis of significant modules. Module–trait relationship heatmap in TCGA-glioma **(A)**, TCGA-GBM **(C)**, CGGA-glioma **(E)**, and CGGA-GBM **(G)**. The row represents the module, and the column represents the trait. The values in the box indicate the correlation and *p*-value. Top 20 enrichment analysis results of the genes in the modules of interest were shown in TCGA-glioma **(B)**, TCGA-GBM **(D)**, CGGA-glioma **(F)**, and CGGA-GBM **(H)**. Most of the enrichment functions are related to immunity.

**Figure 7:**
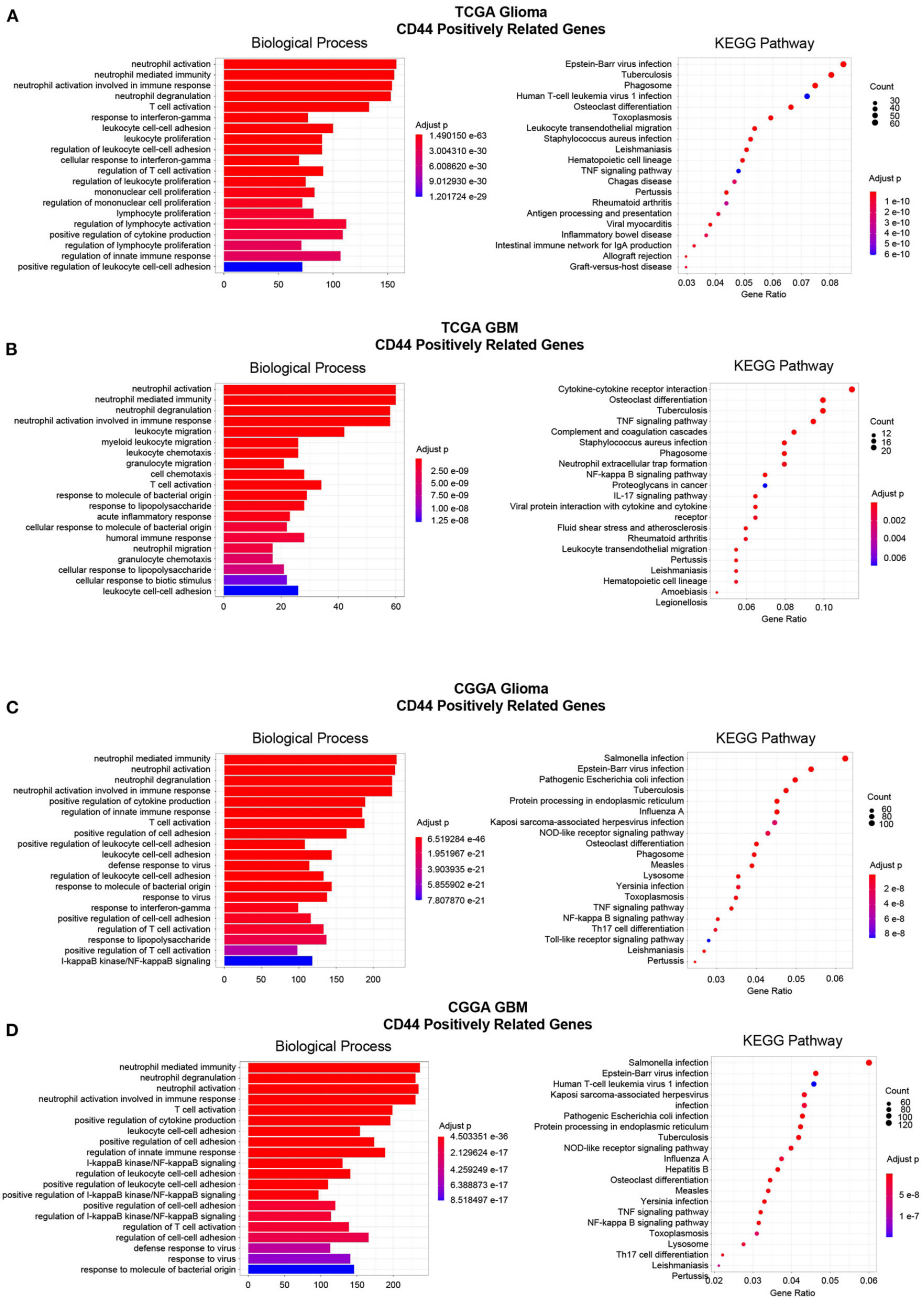
Enrichment analysis of *CD44*-positive related genes. Top 20 enrichment analysis results of *CD44*-positive related genes were showed in TCGA-glioma **(A)**, TCGA-GBM **(B)**, CGGA-glioma **(C)**, and CGGA-GBM **(D)**. Most of the enrichment results are related to immunity.

Then, to gain insight into the particular contribution of *CD44* to glioma immunity, GO and KEGG analyses were used to screen relevant genes in TCGA and CGGA glioma databases. We adopt two different strategies, namely the WGCNA method and the Pearson's correlation method, to obtain the *CD44* relevant genes. First, the general WGCNA analysis flow was taken to divide the genes in different modules and to correlate these module eigengenes with clinical traits including *CD44* expression level. The modules were chosen as they had the highest correlation coefficient with *CD44* transcription level and contained the *CD44* as well. The brown, turquoise, and brown and blue module emerges as the most significant module for TCGA glioma, TCGA GBM, CGGA glioma, and CGGA GBM, respectively (Figures 6A,C,E,G, Supplementary Figure 5). Then, we picked out hub genes in these most significant modules and used them in subsequent enrichment analysis. These hub genes correlated with *CD44* are involved in immune response (e.g., macrophage activation, cytokine production, microglial cell activation, and T cell activation) in both TCGA and CGGA glioma datasets (Figures 6B,D,F,H, Supplementary Table 1). Apart from the positively relevant genes, negatively relevant ones can also help us to understand the function of *CD44*. To acquire *CD44* coexpressed genes in the second Pearson's correlation analysis part, we set different Pearson's |R| value cutoff in TCGA and CGGA glioma datasets as mentioned in the Methods section. Although the enrichment analysis results of these positively correlated genes are also most involved in immunity (Figure 7), we found that some of them are related to angiogenesis, cell adhesion, ECM-receptor interaction, and so on. (Supplementary Tables 2, 3), which is consistent with the former studies (36, 37). On the other hand, the negatively relevant genes of *CD44* take parts in synaptic function (Supplementary Figure 6, Supplementary Tables 2, 3). To sum up, *CD44* plays an important role in glioma immunity.

### Spatial Distribution Preference of *CD44* in GBM

**Figure 8:**
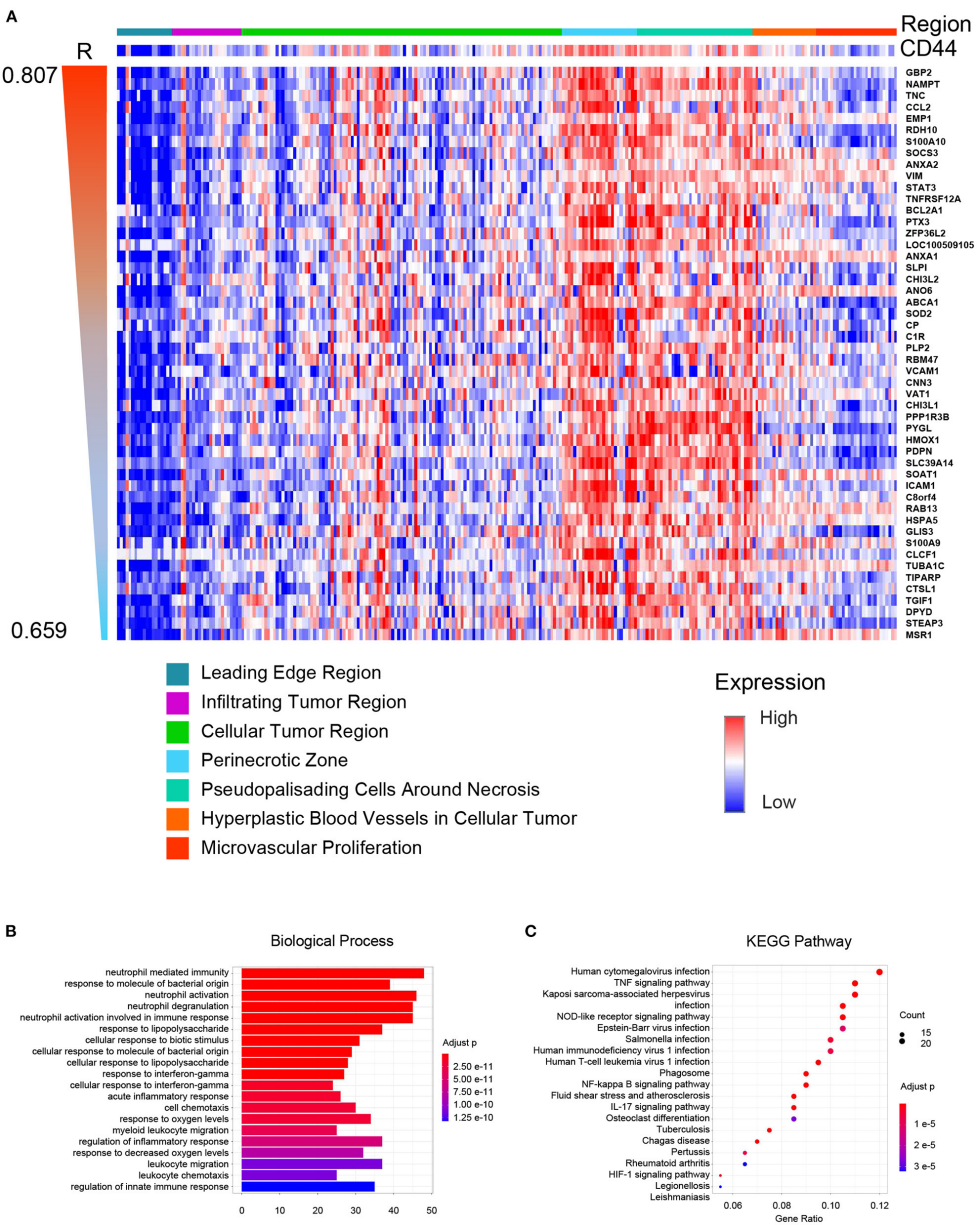
*CD44* spatial expression pattern. **(A)** Heatmap of the top 50 correlated genes based on distribution similarity with *CD44*. The genes were ordered by correlated R index from high to low. Top 20 biological processes **(B)** and top 20 pathways **(C)** of *CD44* spatial expression related genes were shown. The major of enrichment results is about immunity.

Glioblastoma is one of the most heterogeneous tumors, and multiple TCGA transcription subtypes can coexist in the close tumor regions (33). Spatial heterogeneity of glioma is the barrier for treatment. Thus, we compared the *CD44* expression level in different GBM tumor regions, and genes were clustered according to their tumor region features to obtain the expression patterns whose spatial states are highly desirable as *CD44*. The result demonstrates that *CD44* mainly distributes in the perinecrotic region within tumor (Figure 8A). Some of the top correlated genes are immune genes (e.g., *TNC, CCL2, SOCS3, TNFRSF12A, PTX3*, and *VAT1*) (38, 39), invasive genes (e.g., *GBP2, EMP1, VIM, ANO6, RBM47, CHI3L1, HMOX1*, and *ICAM1*) (40–42), proliferative genes (e.g., *ANXA2, ZFP36L2*, and *CHI3L2*) (43, 44), and apoptotic genes (e.g., *SOD2*) (45). From the spatial-based correlated gene pattern, *CD44* may take part in GBM immunity, invasion, and proliferation process. Thus, we further did enrichment analysis of this spatial-based correlated gene pattern to explore underlying biological function of *CD44*. The results confirmed that *CD44* is related to immunity, and invasion (Figures 8B,C, Supplementary Table 4). We also found that *CD44* takes part in response to decreased oxygen levels, HIF-1 signaling pathway, and angiogenesis process (Figures 8B,C, Supplementary Table 4), which is consistent with the spatial distribution preference of perinecrotic zone (Figure 8A). Because *HIF1A* is a regulator of *CD44* and increases *CD44* expression, hypoxic region in breast cancer has also been reported to contain cells with a higher concentration of *CD44* expression (46). In GBM, we uncovered that *CD44*+ cells mainly distribute in hypoxic region with high expression of immune genes.

### Single-Cell Landscape of *CD44* in GBM

**Figure 9:**
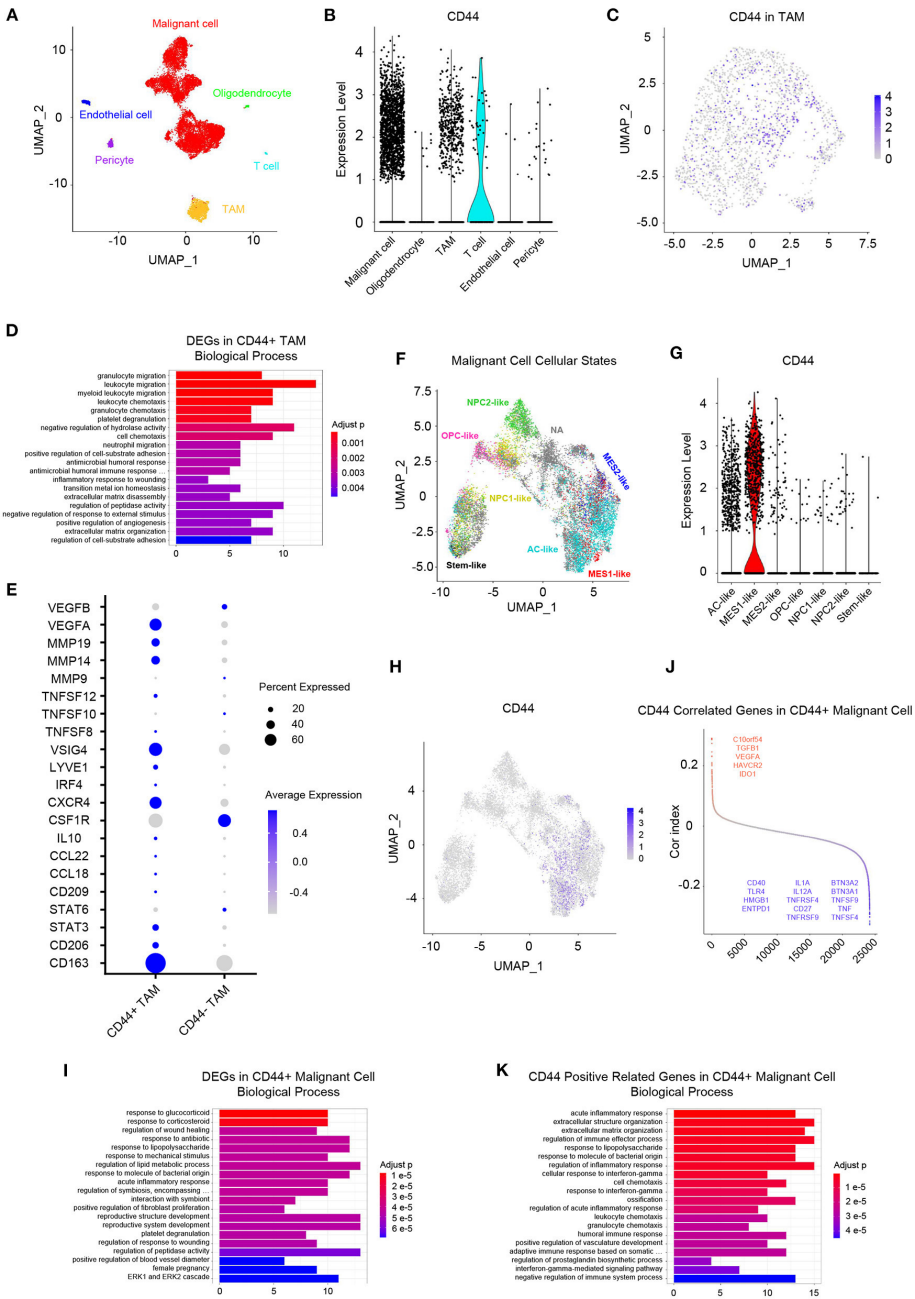
Landscape of *CD44*+ cells in GBM. **(A)** All GBM cell types are projected on UMAP reduction. GBM is composed of malignant tumor cells, oligodendrocytes, TAMs, T cells, endothelial cells, and pericytes. **(B)** Expression level of *CD44* among different GBM cell types. Only malignant cells, TAMs and T cells express *CD44* in GBM. **(C)** Expression of *CD44* in TAMs is projected on UMAP reduction. **(D)** Top 20 biological processes of DEGs in *CD44*+ TAMs compared to *CD44*- TAMs. **(E)** *CD44*+ TAMs express M2-type TAM markers *CD163* and *CD206*. **(F)** UMAP reduction of all six GBM tumor cellular state cells and stem-like cells. **(G)** Expression level of *CD44* among the six GBM tumor cellular state cells and stem-like cells. Only MES1-like tumor cells express *CD44* among different cellular states. **(H)** Expression of *CD44* in tumor cells is projected on UMAP reduction. **(I)** Top 20 biological processes of DEGs in *CD44*+ tumor cells compared to *CD44*- tumor cells. **(J)** *CD44*-correlated genes in *CD44*+ malignant cells. Genes labeled in red are immunosuppressive genes, whose expression level positively correlates with *CD44*; blue colored genes are immune stimulator genes and their expression negatively relates to *CD44*. **(K)** Top 20 biological processes of *CD44* positive-related genes in *CD44*+ malignant cells.

**Figure 10:**
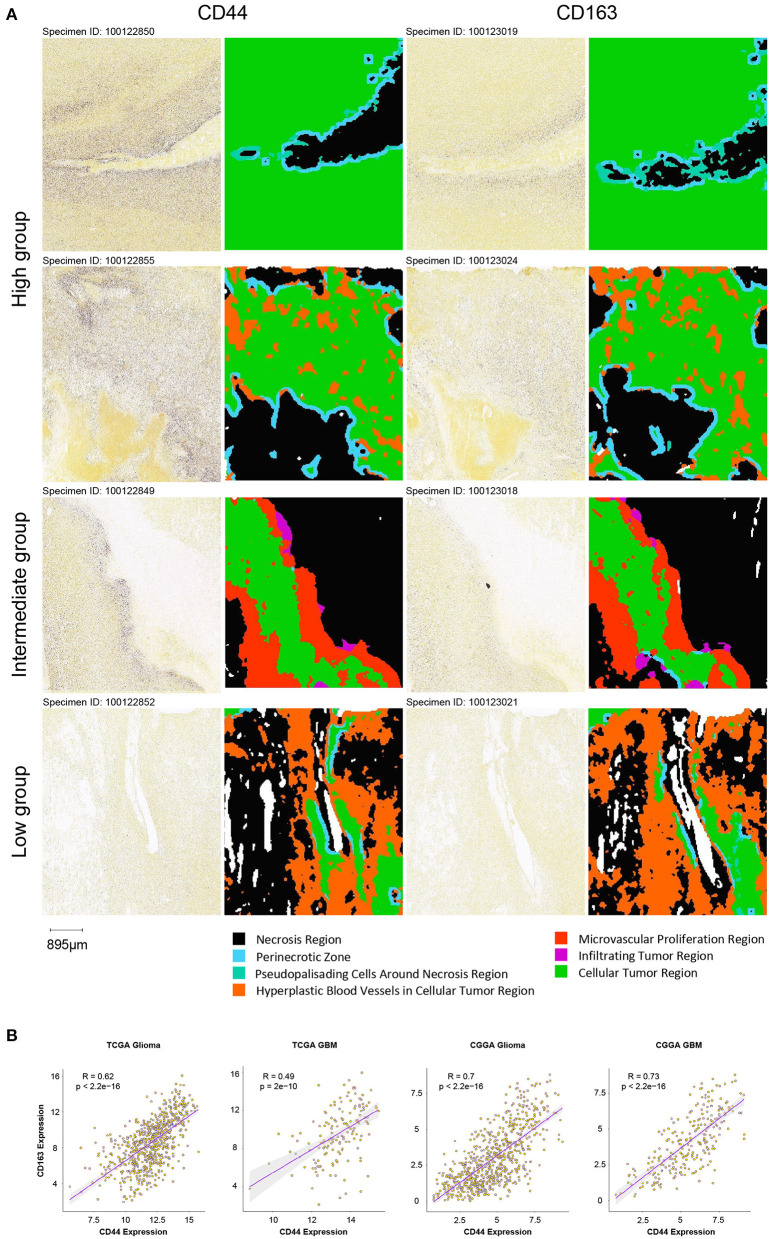
Verification of infiltrating M2 TAMs in different groups. **(A)** Comparison of the infiltration of M2 TAMs (*CD163*+) among the high, intermediate and low intratumoral *CD44*+ cell groups in the IGAP IHC database. Scale, 895 μm. **(B)** Correlation of *CD44* with *CD163* in the TCGA and CGGA glioma datasets.

Whereas, tumor is constituted by malignant cells and surrounding stromal cells, we know few about the heterogeneity of *CD44* transcription in glioma at single-cell resolution until now. Global gene expression however is readily measurable, and the advent of single-cell RNA-seq technology enables us profile individual cell expression within tumors. Therefore, we applied single-cell RNA-seq dataset for GBM (47) to further explore *CD44* expression at single-cell resolution. First, we annotated GBM cells into specific cell types according to CNVs and known cell markers (Supplementary Figures 7B,C). GBM contains malignant tumor cells and TAMs together with a small number of oligodendrocytes, endothelial cells, pericytes, and T cells (Figure 9A). Then, we compared the *CD44* expression level among different cell types and found that only malignant tumor cells, TAMs, and T cells express *CD44* (Figure 9B). TAMs are the major immune cells in GBM, which is in accord with our finding that *CD44* acts in glioma immunity. Because only a part of TAMs express *CD44* (Figure 9C), DEGs between *CD44*+ TAMs and *CD44*- TAMs were calculated and underwent further enrichment analysis. The functional analysis showed that *CD44*+ TAMs are enriched in leukocyte migration, leukocyte chemotaxis, positive regulation of cell-substrate adhesion, extracellular matrix organization, positive regulation of angiogenesis, and so on (Figure 9D, Supplementary Table 5). In addition, *CD44*+ TAMs are in M2-type polarization state presenting with high expression of *CD163, CD206*, and *STAT3*, and they can also activate angiogenesis by secreting *VEGFA* and promote cell invasion through secreting matrix metallopeptidase (e.g., *MMP14, MMP19*) (Figure 9E). Because *CD44* expression level is positively correlated with M2-type macrophage infiltration (Figure 5B), we verified the infiltrating M2 TAMs among different *CD44*+ cell infiltration levels by IHC (Figure 10A) and found that the more *CD44*+ cells are detected, the more M2 TAMs infiltrate, which is consistent with the correlation of *CD44* with *CD163* in the TCGA and CGGA glioma datasets (Figure 10B). By checking expression of *CD44* and *CD163* in glioma samples through IF, *CD44*+*CD163*+ cells were uncovered (Supplementary Figure 8), which confirmed that the *CD44*+ TAMs are in M2 phenotype. We also uncovered that not all T cells express *CD44* (Supplementary Figure 7D), and the enrichment analysis results of *CD44*-related genes in T cells are listed in Supplementary Table 6.

Neftel et al. declare that GBM malignant tumor cells can be grouped into a limited set of cellular states, namely astrocyte-like (AC-like), mesenchymal-1-like (MES1-like), mesenchymal-2-like (MES2-like), oligodendrocyte-progenitor-like (OPC-like), neural-progenitor-1-like (NPC1-like), and neural-progenitor-2-like (NPC2-like) states (32). Because *CD44* was described as a marker of GBM CSCs, also known as glioma stem–progenitor cells or glioma-initiating cells (7), we also annotated the stem-like cells besides the six GBM tumor cellular states in malignant cells (Figure 9F). Furthermore, we revealed MES1-like state preference of *CD44* (Figure 9G), which is similar to TCGA-mesenchymal subtype preference of *CD44* (Figure 3). Surprisingly, no GBM stem-like cells express *CD44*, suggesting that it is not a suitable CSC marker (Figure 9G). Next, we compared the DEGs between *CD44*+ tumor cells and *CD44*- tumor cells (Figure 9H), and the enrichment analysis results verified the immunity function of *CD44* (Figure 9I, Supplementary Table 7). In addition, we calculated the Pearson's correlated genes with *CD44* in *CD44*+ tumor cells and uncovered that some positive related genes are immunosuppressive genes (e.g., *C10orf54, TGFB1, VEGFA, HAVCR2, and IDO1*) and that some negative related genes are immune stimulator genes (e.g., *BTN3A2, BTN3A1, TNFSF9, TNF, TNFSF4, IL1A, IL12A, TNFRSF4, CD27, TNFRSF9, CD40, TLR4, HMGB1, and ENTPD1*) (Figure 9J, Supplementary Table 8). The enrichment analysis results of *CD44*-positive related genes in *CD44*+ malignant cell showed that *CD44* correlates with immune activities as well (Figure 9K, Supplementary Table 7).

### Correlation of *CD44* and GBM Immunosuppression

**Figure 11:**
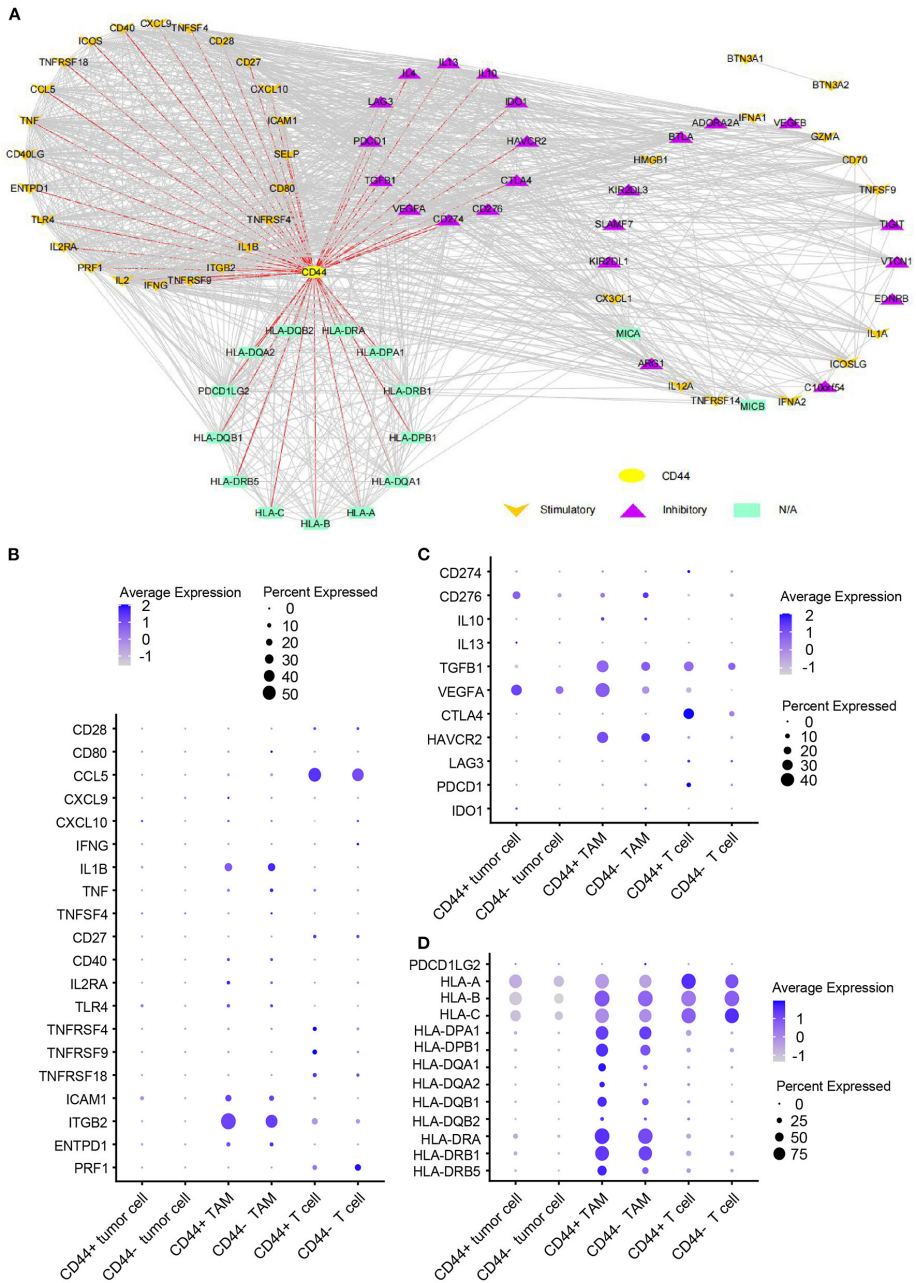
Expression of immunomodulators in *CD44*+ glioma cells. **(A)** Protein–protein interaction network of *CD44* and immunomodulators. The direct interaction between *CD44* and immunomodulators was colored in red. Expression dot plot of stimulatory immunomodulators with direct interaction to *CD44* **(B)**, inhibitory immunomodulators with direct interaction to *CD44* **(C)** and other immunomodulators with direct interaction to *CD44* **(D)** among tumor cells, TAMs, and T cells.

**Figure 12:**
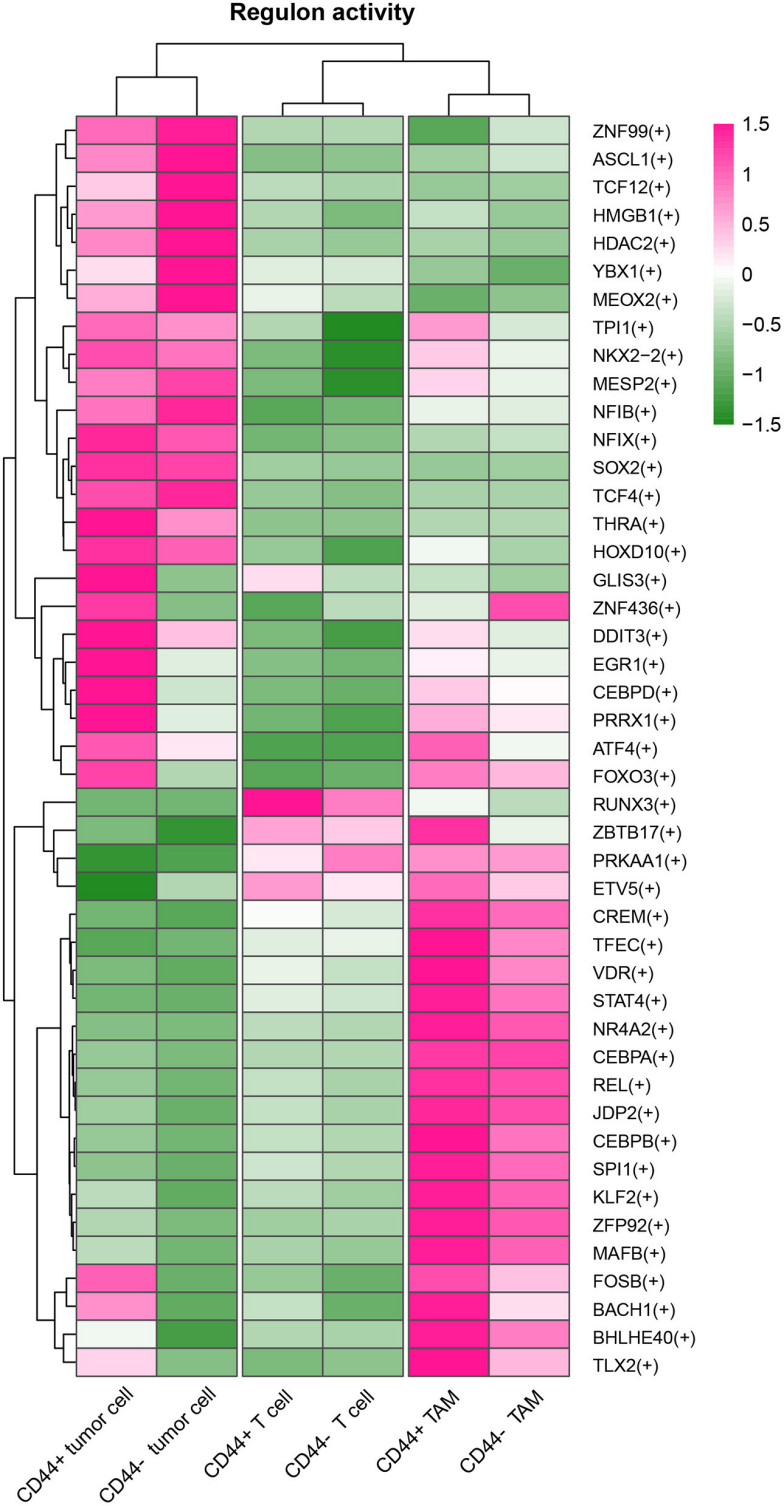
Heatmap of regulon activity among tumor cells, TAMs and T cells. The cell types and activated regulons were clustered respectively.

*CD44* is related to immunity in glioma, *CD44*+ malignant tumor cells are in MES-1-like state, and *CD44*+ TAMs are in M2 phenotype. Therefore, we hypothesized that glioma *CD44*+ cells take part in tumor immunosuppression. First, we used the immunomodulators (21) with *CD44* to construct the protein–protein interaction network, and the immunomodulators with direct interaction to CD44 were identified (Figure 11A). Then, we found that some of the *CD44*-correlated genes in *CD44*+ cells are immunomodulators (Figure 9J, Supplementary Figures 7E,F). For example, in *CD44*+ T cells, some positive related genes are immunosuppressive genes (e.g., *CD274, VEGFA, PDCD1*, and *CTLA4*) and that some negative related genes are immune stimulator genes (e.g., *CCL5, ITGB2, ICAM1*, and *PRF1*). Thus, *CD44*+ malignant tumor cells, *CD44*+ TAMs, and *CD44*+ T cells may induce immunosuppression in glioma. Next, we explored the expression of the immunomodulators with direct interaction to *CD44* among the glioma *CD44*+ cells (Figures 11B–D). Each *CD44*+ cell-type functions immunosuppression through different ways: *CD44*+ tumor cells express *CD276, IL13, VEGFA*, and *IDO1*; *CD44*+ TAMs express *IL10, TGFB1, VEGFA*, and *HAVCR2*; *CD44*+ T cells express *CD274, TGFB1, CTLA4, LAG3*, and *PDCD1* (Figure 11C). To our surprise, we found that *CD44*+ T cells express both *CD274* (namely *PD-L1*) and *PDCD1* (namely *PD-1*) in glioma. In addition, we built the regulon network of glioma cells, and the regulon network of *CD44*+ cells is different from *CD44*- cells (Figure 12). *DDIT3* regulon is upregulated in *CD44*+ tumor cells, which can inhibit type I interferon (*IFN-I*) and *IFN*-stimulated gene production (48). *RUNX3* is upregulated in *CD44*+ T cells, which serves as immunosuppressive role in tumor (49). Therefore, *CD44*+ glioma cells participate in glioma immunosuppression.

**Figure 13:**
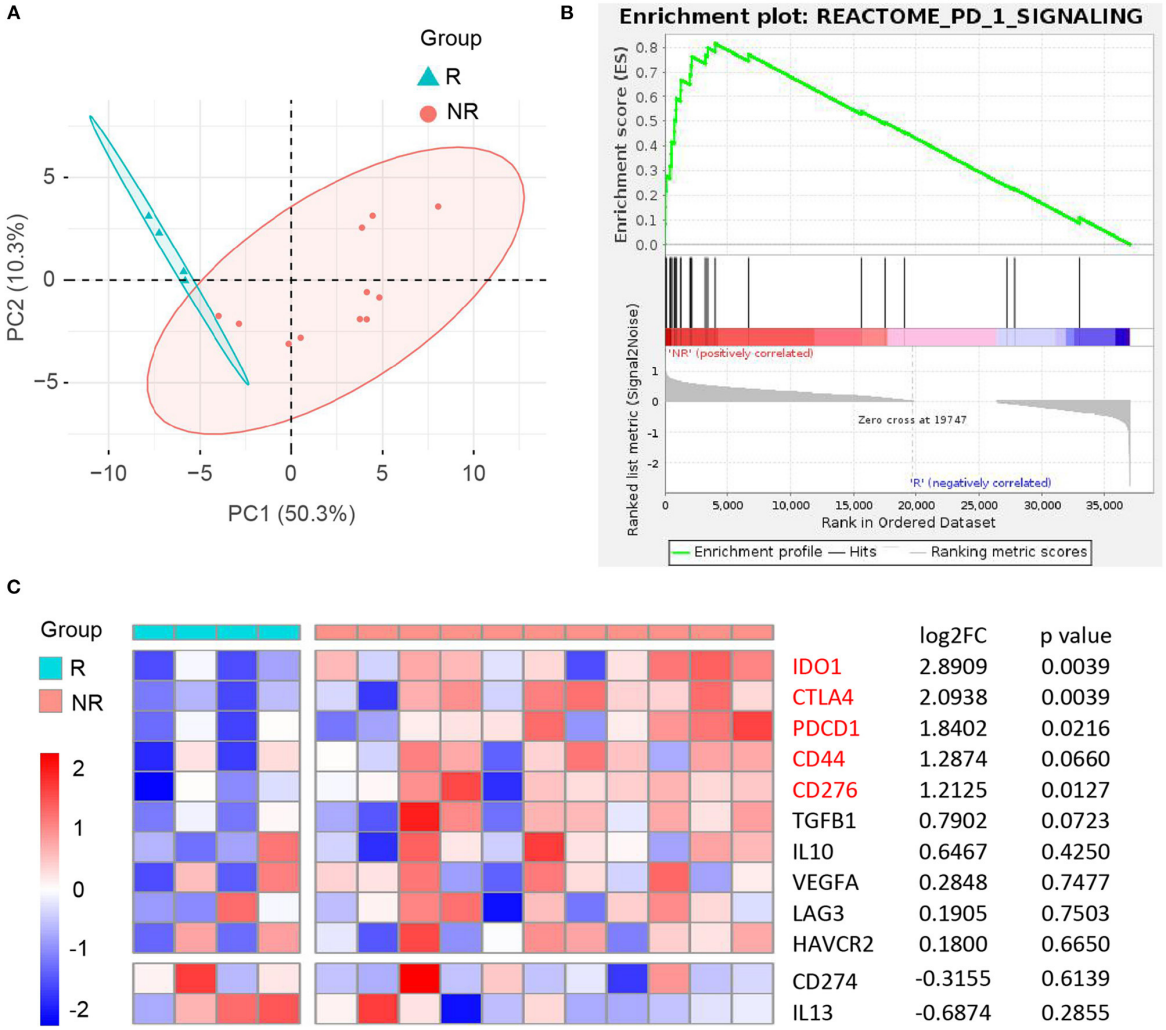
Correlation between *CD44* and anti-*PD-1* immunotherapy in recurrent GBM. **(A)** Principal component analysis of responder (R) and nonresponder (NR) recurrent GBM treated with anti-*PD-1* immunotherapy based on RNA-seq data. **(B)** NR subgroup is enriched in REACTOME *PD-1* signaling pathway compared to R subgroup. **(C)** Heatmap of *CD44* and its directly interacted inhibitory immunomodulators expression between R and NR recurrent GBM subgroups. NR group was compared to R group, and the matching log2FC and *p*-value of each gene were shown in the right column.

It is an ongoing endeavor to conduct researches on the development of new drugs, and new strategies eradicating cancer target the immune cells with immune checkpoints (50). *PD-1* blockade yielded promising results in many cancers, and the Ivy Consortium initiated a multiinstitution, randomized, open-label pilot study of pembrolizumab, an anti-*PD-1* monoclonal antibody, in patients with recurrent GBM (51). To uncover the correlation between immunotherapy and *CD44* in glioma, we extracted bulk RNA-seq data of patients with recurrent GBM receiving adjuvant, postsurgical *PD-1* blockade therapy from this study. The responder and nonresponder patients are different in mRNA expression pattern based on principal component analysis (Figure 13A). Furthermore, the patients with nonresponder recurrent GBM are enriched in *PD-1* signaling pathway, suggesting that they have immunosuppression microenvironment and would display therapy resistance (Figure 13B). *CD44* and most of its directly interacted inhibitory immunomodulators express relatively higher in immunotherapy nonresponder subgroup than responder subgroup, although some genes are not statistically significant (Figure 13C). These results confirm immunosuppression role of *CD44* in glioma.

## Discussion

In this study, the function of *CD44* was explored in glioma at bulk, spatial, and single-cell level, respectively. We discovered the immune characteristic of *CD44* in glioma, which plays a critical role in clinical outcomes.

One meta-analysis claims higher expression of *CD44* that predicts poor survival in glioma particularly in those with WHO grades II and III glioma (52), and another clinical research discovers that higher tumor expression of *CD44* acts as a negative prognosis indicator in patients with GBM (53). Our work also found that *CD44* can be a prognostic biomarker, whose transcription level is related to malignancy glioma and poor prognosis in two notable glioma cohorts (11, 12). *CD44* is a cell membrane glycoprotein which involved in diverse tumor aggressive processes including invasion, proliferation, apoptosis, and angiogenesis (37), but there are no studies reporting its activity in glioma immunity. To gain the overview of *CD44* function, we adopted multiple methods to obtain *CD44*-related genes, including WGCNA, the Pearson's correlation, and DEGs. Later, we did the enrichment analysis of these gene sets of interest to get further understanding of them. It is very interesting to notice that immunity function is more significant than *CD44* other functions (Figures 6, 7, Supplementary Tables 1–3), such as invasion, proliferation, and angiogenesis reported as before. Furthermore, *CD44* transcription level is related to M2 macrophage infiltration at bulk-seq resolution (Figure 5B), and this finding is verified by single-cell RNA-seq data that *CD44*+ TAMs are in M2-type state (Figure 9E) and by IHC that glioma with higher *CD44*+ cells infiltration has more infiltrating M2 macrophages (Figure 10A). In addition, *CD44*+*CD163*+ cells were verified by IF on human glioma samples (Supplementary Figure 8). M2 TAM accelerating tumorigenicity (54) is one of the underlying reasons why *CD44* can be the convincing prognostic biomarker for glioma. These results support that *CD44* is a new biomarker for M2 TAMs.

In glioma mouse model, *CD44* was restricted to hypoxic and perivascular tumor regions, and *HIF-2*α, a hypoxia signature, was correlated with *CD44* in human glioma (55). We also checked the spatial preference of *CD44* in glioma and found that CD44 distributes in the perinecrotic zone within tumor (Figure 8A). The correlated gene patterns based on *CD44* distribution preference are involved in hypoxia response and angiogenesis which coincides with the former research, but we found that the major of enrichment analysis results are immune-related activities that highlight its immune function (Figures 8B,C). Apart from *CD44* spatial preference, transcription subtype preference, namely mesenchymal subtype, in bulk-seq level (Figure 3) and cellular state preference, namely MES1-like state, at single-cell resolution (Figure 9G) are also revealed by our study. Whereas, TCGA mesenchymal subtype GBM is characterized as immunosuppressive and therapy resistant tumor (33), the significance of *CD44* expression preference in specific GBM molecular subtype and tumor cell cellular state is unclear. Therefore, we calculated the DEGs between *CD44*+ tumor cells and *CD44*- tumor cells and identified the Pearson's correlated genes in *CD44*+ tumor cells (Figures 9H–K). These results support our new finding that *CD44* acts in glioma immunity. The major of glioma-infiltrating immune cells are TAMs, and an increased infiltering TAM correlates with shorter survival time (35). Thus, the surface markers of TAMs may be the potential therapeutic targets. TAMs in glioma are commonly identified by the expression of *CD11b, CD14, CD68, CD163*, and *CD206*. Additionally, we demonstrated that *CD44* expression has high concordance with *CD11b, CD14, CD68, CD163*, and *CD206*, which indicates a synergistic relationship between *CD44* and macrophage markers in glioma (Supplementary Figure 9). This reminds us that for patients who acquired resistance to standard therapy based on the expression of *CD44* (36), we should pay attention to the increasing of TAM makers and arising of M2-type TAMs infiltrating.

To go a step further, we explored the immunosuppressive role of *CD44* in glioma. As reported, *CD44* expression level is positively correlated with *PD-L1, PD-1, IL10*, and *TGFB1* expression in some cancers (56, 57). Thus, coexpression of *CD44* and checkpoint family members were checked in glioma, and we found that *CD44* expression positively correlates with the expression level of *PD-L1* and *PD-1* and correlates with the expression level of *PDCD1LG2* (namely *PD-L2*) as well in the TCGA and CGGA glioma datasets (Supplementary Figure 10). *HAVCR2* plays almost the same immunosuppressive functions as *PD-1* in glioma (58), and we also found that *CD44* shows a consistent correlation with *HAVCR2* and *PD-1* in glioma (Supplementary Figure 10). It has been reported that *CD40, TNFSF14, LGALS9*, and *SIGLEC10* high expression levels are associated with glioma malignancy grade and negative prognosis (59–61). Therefore, there are some positive relationships between *CD44* and these immune checkpoints in glioma (Supplementary Figure 10). However, further analysis found that *NCR3LG1* shows negative relationship with *CD44* in glioma (Supplementary Figure 10), which may be partially explained by the low expression in malignant glioma and positive glioma survival biomarker of *NCR3LG1* (Supplementary Figure 11). *CD44* can positively regulate the expression of *PD-L1* by activating *PD-L1* transcription partly *via* the association between its intracytoplasmic domain and a regulatory region in *PD-L1* (62), and *CD44*+ tumor-initiating cells in head and neck squamous cell carcinoma suppress antitumor immunity through inducible expression of *PD-L1* (63). *PD-L1* represses antitumor immunity through its interaction with the *PD-1* receptor on T cells (64). Furthermore, *PD-1* is obviously elevated in *CD44*+ T cell than *CD44*- T cell (65). *CD44* takes part in formation and persistence of regulatory T cells which play a vital role in tumor immunosuppression: *LGALS9* interacts with *CD44* in association with *TGF-*β receptors to drive *FOXP3* expression in regulatory T cells (66); *CD44* maintains *FOXP3*+ regulatory T cell persistence *via* inducing production of *IL10* and *TGFB1* (67). Conversely, other immunomodulators can also regulate *CD44* expression levels. *IL4* and *IL13* can upregulate *CD44* expression in human cervical adenocarcinoma cell lines and colonic epithelial cell lines (68, 69). Our results revealed that distinct *CD44*+ cell type plays the immunosuppressive role through expressing different immunomodulators in GBM, and it is interesting to uncover that *CD44*+ T cells express both *PD-L1* and *PD-1* (Figure 11C). Thus, glioma-infiltrating *CD44*+ T cells can inhibit antitumor immunity by inducible expression of *PD-L1*. Finally, the correlation of *CD44* mRNA expression and glioma immunotherapy was studied. *CD44* and its directly interacted inhibitory immunomodulators are upregulated in patients with nonresponder recurrent GBM receiving *PD-1* blockade therapy. However, it still needs more clinical studies to confirm the relationship between *CD44* and glioma immunotherapy for the small number of glioma samples treated with immunotherapy.

## Conclusion

Our research limitations include analysis from publicly available data cohorts and limited data interpretation by the lack of mechanistic approaches and causality association because this study does not implement experiments *in vivo*. The finding of a new candidate biomarker *CD44* for M2 TAMs and its immunosuppressive function in glioma is hypothesis-generating and needs more wet experiment validation. Future works needed to advance that this field further should focus on exploring downstream pathways through which *CD44* acts its immunity role in different glioma cell types, such as tumor cells, TAMs, and T cells.

## Data Availability Statement

Publicly available datasets were analyzed in this study. These data can be found at: TCGA database (https://www.cancer.gov/about-nci/organization/ccg/research/structural-genomics/tcga), the CGGA database (http://www.cgga.org.cn/), the IGAP database (http://glioblastoma.alleninstitute.org/), and the GEO database (https://www.ncbi.nlm.nih.gov/geo) with accession number GSE121810 and GSE103224.

## Author Contributions

YX, HX, and HL conceptualized and coordinated the work. YX prepared the acquisition, analysis, and interpretation of data. YX and KY prepared the figures and tables. YX, ZW, MZ, YD, and WJ wrote the original draft. YZ, CQ, YL, HX, and HL reviewed and edited the manuscript. All authors have read and approved the final version of manuscript.

## Funding

The research work was supported by grants from the National Natural Science Foundation of China (81972350), Jiangsu Science and Education Strengthening Engineering Innovation Team Project (CXTDA2017050), Medical Research Foundation of Jiangsu Health Commission (H2019059), Medical Science and Technology Development Foundation of Nanjing (ZDX16011) and Postgraduate Research and Practice Innovation Program of Jiangsu Province (SJCX19_0331 and KYCX20_1419).

## Conflict of Interest

The authors declare that the research was conducted in the absence of any commercial or financial relationships that could be construed as a potential conflict of interest.

## Publisher's Note

All claims expressed in this article are solely those of the authors and do not necessarily represent those of their affiliated organizations, or those of the publisher, the editors and the reviewers. Any product that may be evaluated in this article, or claim that may be made by its manufacturer, is not guaranteed or endorsed by the publisher.

## Abbreviations

CGGA: the Chinese Glioma Genome Atlas
CI: confidence interval
CSCs: cancer stem cells
DEGs: differentially expressed genes
GBM: glioblastoma
GO: gene ontology
HR: hazard ratio
IGAP: the Ivy Glioblastoma Atlas Project
KEGG: Kyoto Encyclopedia of Genes and Genomes
LGG: low-grade glioma
OS: overall survival
TAMs: tumor-associated macrophages
TCGA: the Cancer Genome Atlas
WGCNA: weighted gene coexpression network analysis.

## References

[1] Lapointe S Perry A Butowski NA. Primary brain tumours in adults. Lancet. (2018) 392:432–46. 10.1016/S0140-6736(18)30990-5 30060998

[2] Grossman SA Ye X Piantadosi S Desideri S Nabors LB Rosenfeld M. Survival of patients with newly diagnosed glioblastoma treated with radiation and temozolomide in research studies in the United States. Clin Cancer Res. (2010) 16:2443–9. 10.1158/1078-0432.CCR-09-3106 20371685 PMC2861898

[3] Chinot OL Wick W Mason W Henriksson R Saran F Nishikawa R. Bevacizumab plus radiotherapy-temozolomide for newly diagnosed glioblastoma. N Engl J Med. (2014) 370:709–22. 10.1056/NEJMoa1308345 24552318

[4] Brown MP Ebert LM Gargett T. Clinical chimeric antigen receptor-T cell therapy: a new and promising treatment modality for glioblastoma. Clin Transl Immunology. (2019) 8:e1050. 10.1002/cti2.1050 31139410 PMC6526894

[5] Xu H Niu M Yuan X Wu K Liu A. CD44 as a tumor biomarker and therapeutic target. Exp Hematol Oncol. (2020) 9:36. 10.1186/s40164-020-00192-0 33303029 PMC7727191

[6] Chen C Zhao S Karnad A Freeman JW. The biology and role of CD44 in cancer progression: therapeutic implications. J Hematol Oncol. (2018) 11:64. 10.1186/s13045-018-0605-5 29747682 PMC5946470

[7] Dzobo K Senthebane DA Ganz C Thomford NE Wonkam A Dandara C. Advances in therapeutic targeting of cancer stem cells within the tumor microenvironment: an updated review. Cells. (2020) 9:1896. 10.3390/cells9081896 32823711 PMC7464860

[8] Hou C Ishi Y Motegi H Okamoto M Ou Y Chen J. Overexpression of CD44 is associated with a poor prognosis in grade II/III gliomas. J Neurooncol. (2019) 145:201–10. 10.1007/s11060-019-03288-8 31506754

[9] Gudbergsson JM Christensen E Kostrikov S Moos T Duroux M Kjaer A. Conventional treatment of glioblastoma reveals persistent CD44(+) subpopulations. Mol Neurobiol. (2020) 57:3943–55. 10.1007/s12035-020-02004-2 32632605

[10] Ponta H Sherman L Herrlich PA. CD44: from adhesion molecules to signalling regulators. Nat Rev Mol Cell Biol. (2003) 4:33–45. 10.1038/nrm1004 12511867

[11] Cancer Genome Atlas Research N. Comprehensive genomic characterization defines human glioblastoma genes and core pathways. Nature. (2008) 455:1061–8. 10.1038/nature07385 18772890 PMC2671642

[12] Zhao Z Meng F Wang W Wang Z Zhang C Jiang T. Comprehensive RNA-seq transcriptomic profiling in the malignant progression of gliomas. Sci Data. (2017) 4:170024. 10.1038/sdata.2017.24 28291232 PMC5349247

[13] Louis DN Perry A Reifenberger G von Deimling A Figarella-Branger D Cavenee WK. The 2016 world health organization classification of tumors of the central nervous system: a summary. Acta Neuropathol. (2016) 131:803–20. 10.1007/s00401-016-1545-1 27157931

[14] Puchalski RB Shah N Miller J Dalley R Nomura SR Yoon JG. An anatomic transcriptional atlas of human glioblastoma. Science. (2018) 360:660–3. 10.1126/science.aaf2666 29748285 PMC6414061

[15] Yoshihara K Shahmoradgoli M Martinez E Vegesna R Kim H Torres-Garcia W. Inferring tumour purity and stromal and immune cell admixture from expression data. Nat Commun. (2013) 4:2612. 10.1038/ncomms3612 24113773 PMC3826632

[16] Aran D Hu Z Butte AJ. xCell: digitally portraying the tissue cellular heterogeneity landscape. Genome Biol. (2017) 18:220. 10.1186/s13059-017-1349-1 29141660 PMC5688663

[17] Newman AM Liu CL Green MR Gentles AJ Feng W Xu Y. Robust enumeration of cell subsets from tissue expression profiles. Nat Methods. (2015) 12:453–7. 10.1038/nmeth.3337 25822800 PMC4739640

[18] Langfelder P Horvath S. WGCNA: an R package for weighted correlation network analysis. BMC Bioinformatics. (2008) 9:559. 10.1186/1471-2105-9-559 19114008 PMC2631488

[19] Huang da W Sherman BT Lempicki RA. Bioinformatics enrichment tools: paths toward the comprehensive functional analysis of large gene lists. Nucleic Acids Res. (2009) 37:1–13. 10.1093/nar/gkn923 19033363 PMC2615629

[20] Kanehisa M Sato Y Kawashima M Furumichi M Tanabe M. KEGG as a reference resource for gene and protein annotation. Nucleic Acids Res. (2016) 44:D457–62. 10.1093/nar/gkv1070 26476454 PMC4702792

[21] Thorsson V Gibbs DL Brown SD Wolf D Bortone DS Ou Yang TH. The Immune Landscape of Cancer. Immunity. (2018) 48:812–30.e814. 10.1016/j.immuni.2018.03.023 29628290 PMC5982584

[22] Love MI Huber W Anders S. Moderated estimation of fold change and dispersion for RNA-seq data with DESeq2. Genome Biol. (2014) 15:550. 10.1186/s13059-014-0550-8 25516281 PMC4302049

[23] Barrett T Troup DB Wilhite SE Ledoux P Rudnev D Evangelista C. NCBI GEO: mining tens of millions of expression profiles–database and tools update. Nucleic Acids Res. (2007) 35:D760–765. 10.1093/nar/gkl887 17099226 PMC1669752

[24] Stuart T Butler A Hoffman P Hafemeister C Papalexi E Mauck WM 3rd. Comprehensive integration of single-cell data. Cell. (2019) 177:1888–902.e1821. 10.1016/j.cell.2019.05.031 31178118 PMC6687398

[25] Aibar S Gonzalez-Blas CB Moerman T Huynh-Thu VA Imrichova H Hulselmans G. SCENIC: single-cell regulatory network inference and clustering. Nat Methods. (2017) 14:1083–6. 10.1038/nmeth.4463 28991892 PMC5937676

[26] Qiu X Mao Q Tang Y Wang L Chawla R Pliner HA. Reversed graph embedding resolves complex single-cell trajectories. Nat Methods. (2017) 14:979–82. 10.1038/nmeth.4402 28825705 PMC5764547

[27] Huang LE. Friend or foe-IDH1 mutations in glioma 10 years on. Carcinogenesis. (2019) 40:1299–307. 10.1093/carcin/bgz134 31504231 PMC6875900

[28] Ren X Cui X Lin S Wang J Jiang Z Sui D. Co-deletion of chromosome 1p/19q and IDH1/2 mutation in glioma subsets of brain tumors in Chinese patients. PLoS ONE. (2012) 7:e32764. 10.1371/journal.pone.0032764 22427879 PMC3299680

[29] Zhao J Ma W Zhao H. Loss of heterozygosity 1p/19q and survival in glioma: a meta-analysis. Neuro Oncol. (2014) 16:103–12. 10.1093/neuonc/not145 24311641 PMC3870828

[30] Toms SA Kim CY Nicholas G Ram Z. Increased compliance with tumor treating fields therapy is prognostic for improved survival in the treatment of glioblastoma: a subgroup analysis of the EF-14 phase III trial. J Neurooncol. (2019) 141:467–73. 10.1007/s11060-018-03057-z 30506499 PMC6342854

[31] Filbin MG Tirosh I Hovestadt V Shaw ML Escalante LE Mathewson ND. Developmental and oncogenic programs in H3K27M gliomas dissected by single-cell RNA-seq. Science. (2018) 360:331–5. 10.1126/science.aao4750 29674595 PMC5949869

[32] Neftel C Laffy J Filbin MG Hara T Shore ME Rahme GJ. An integrative model of cellular states, plasticity, and genetics for glioblastoma. Cell. (2019) 178:835–49.e821. 10.1016/j.cell.2019.06.024 31327527 PMC6703186

[33] Wang Q Hu B Hu X Kim H Squatrito M Scarpace L. Tumor evolution of glioma-intrinsic gene expression subtypes associates with immunological changes in the microenvironment. Cancer Cell. (2018) 33:152. 10.1016/j.ccell.2017.12.012 29316430 PMC5892424

[34] Ceccarelli M Barthel FP Malta TM Sabedot TS Salama SR Murray BA. Molecular profiling reveals biologically discrete subsets and pathways of progression in diffuse glioma. Cell. (2016) 164:550–63. 10.1016/j.cell.2015.12.028 26824661 PMC4754110

[35] Ye J Yang Y Jin J Ji M Gao Y Feng Y. Targeted delivery of chlorogenic acid by mannosylated liposomes to effectively promote the polarization of TAMs for the treatment of glioblastoma. Bioact Mater. (2020) 5:694–708. 10.1016/j.bioactmat.2020.05.001 32478203 PMC7248290

[36] Mooney KL Choy W Sidhu S Pelargos P Bui TT Voth B. The role of CD44 in glioblastoma multiforme. J Clin Neurosci. (2016) 34:1–5. 10.1016/j.jocn.2016.05.012 27578526

[37] Chen D Li D Xu XB Qiu S Luo S Qiu E. Galangin inhibits epithelial-mesenchymal transition and angiogenesis by downregulating CD44 in glioma. J Cancer. (2019) 10:4499–508. 10.7150/jca.31487 31528214 PMC6746128

[38] Locatelli M Ferrero S Martinelli Boneschi F Boiocchi L Zavanone M Maria Gaini S. The long pentraxin PTX3 as a correlate of cancer-related inflammation and prognosis of malignancy in gliomas. J Neuroimmunol. (2013) 260:99–106. 10.1016/j.jneuroim.2013.04.009 23664694

[39] Yang P Wang K Zhang C Wang Z Liu Q Wang J. Novel roles of VAT1 expression in the immunosuppressive action of diffuse gliomas. Cancer Immunol Immunother. (2021) 70:2589–600. 10.1007/s00262-021-02865-z 33576871 PMC10992787

[40] Wang J Li X Wu H Wang H Yao L Deng Z. EMP1 regulates cell proliferation, migration, and stemness in gliomas through PI3K-AKT signaling and CD44. J Cell Biochem. (2019) 120:17142–50. 10.1002/jcb.28974 31111534

[41] Xuan ZB Wang YJ Xie J. ANO6 promotes cell proliferation and invasion in glioma through regulating the ERK signaling pathway. Onco Targets Ther. (2019) 12:6721–31. 10.2147/OTT.S211725 31692479 PMC6708391

[42] Yu S Yu X Sun L Zheng Y Chen L Xu H. GBP2 enhances glioblastoma invasion through Stat3/fibronectin pathway. Oncogene. (2020) 39:5042–55. 10.1038/s41388-020-1348-7 32518375

[43] Chen L Lin L Xian N Zheng Z. Annexin A2 regulates glioma cell proliferation through the STAT3cyclin D1 pathway. Oncol Rep. (2019) 42:399–413. 10.3892/or.2019.7155 31115554

[44] Che Mat MF Mohamad Hanif EA Abdul Murad NA Ibrahim K Harun R Jamal R. Silencing of ZFP36L2 increases sensitivity to temozolomide through G2/M cell cycle arrest and BAX mediated apoptosis in GBM cells. Mol Biol Rep. (2021) 48:1493–503. 10.1007/s11033-021-06144-z 33590411

[45] Cheng Y Dai C Zhang J. SIRT3-SOD2-ROS pathway is involved in linalool-induced glioma cell apoptotic death. Acta Biochim Pol. (2017) 64:343–50. 10.18388/abp.2016_1438 28567457

[46] Krishnamachary B Penet MF Nimmagadda S Mironchik Y Raman V Solaiyappan M. Hypoxia regulates CD44 and its variant isoforms through HIF-1alpha in triple negative breast cancer. PLoS ONE. (2012) 7:e44078. 10.1371/journal.pone.0044078 22937154 PMC3429433

[47] Yuan J Levitin HM Frattini V Bush EC Boyett DM Samanamud J. Single-cell transcriptome analysis of lineage diversity in high-grade glioma. Genome Med. (2018) 10:57. 10.1186/s13073-018-0567-9 30041684 PMC6058390

[48] Wang S Hou P Pan W He W He DC Wang H. DDIT3 targets innate immunity *via* the DDIT3-OTUD1-MAVS pathway to promote bovine viral diarrhea virus replication. J Virol. (2021) 95:e02351–20. 10.1128/JVI.02351-20 33361422 PMC8094964

[49] Manandhar S Lee YM. Emerging role of RUNX3 in the regulation of tumor microenvironment. BMB Rep. (2018) 51:174–81. 10.5483/BMBRep.2018.51.4.033 29429451 PMC5933212

[50] Akhavan D Alizadeh D Wang D Weist MR Shepphird JK Brown CE. CAR T cells for brain tumors: Lessons learned and road ahead. Immunol Rev. (2019) 290:60–84. 10.1111/imr.12773 31355493 PMC6771592

[51] Cloughesy TF Mochizuki AY Orpilla JR Hugo W Lee AH Davidson TB. Neoadjuvant anti-PD-1 immunotherapy promotes a survival benefit with intratumoral and systemic immune responses in recurrent glioblastoma. Nat Med. (2019) 25:477–86. 10.1038/s41591-018-0337-7 30742122 PMC6408961

[52] Wu G Song X Liu J Li S Gao W Qiu M. Expression of CD44 and the survival in glioma: a meta-analysis. Biosci Rep. (2020) 40:BSR20200520. 10.1042/BSR20200520 32232385 PMC7160241

[53] Si D Yin F Peng J Zhang G. High Expression of CD44 Predicts a Poor Prognosis in Glioblastomas. Cancer Manag Res. (2020) 12:769–75. 10.2147/CMAR.S233423 32099472 PMC7006859

[54] Myers KV Pienta KJ Amend SR. Cancer Cells and M2 Macrophages: Cooperative Invasive Ecosystem Engineers. Cancer Control. (2020) 27:1073274820911058. 10.1177/1073274820911058 32129079 PMC7066590

[55] Johansson E Grassi ES Pantazopoulou V Tong B Lindgren D Berg TJ. CD44 Interacts with HIF-2alpha to Modulate the Hypoxic Phenotype of Perinecrotic and Perivascular Glioma Cells. Cell Rep. (2017) 20:1641–53. 10.1016/j.celrep.2017.07.049 28813675

[56] Zhang C Wang H Wang X Zhao C Wang H. CD44, a marker of cancer stem cells, is positively correlated with PD-L1 expression and immune cells infiltration in lung adenocarcinoma. Cancer Cell Int. (2020) 20:583. 10.1186/s12935-020-01671-4 33372595 PMC7720536

[57] Dzobo K Sinkala M. Cancer Stem Cell Marker CD44 Plays Multiple Key Roles in Human Cancers: Immune Suppression/Evasion, Drug Resistance, Epithelial-Mesenchymal Transition, and Metastasis. OMICS. (2021) 25:313–32. 10.1089/omi.2021.0025 33961518

[58] Li G Wang Z Zhang C Liu X Cai J Wang Z. Molecular and clinical characterization of TIM-3 in glioma through 1,024 samples. Oncoimmunology. (2017) 6:e1328339. 10.1080/2162402X.2017.1328339 28919992 PMC5593703

[59] Liang T Wang X Wang F Feng E You G. Galectin-9: A Predictive Biomarker Negatively Regulating Immune Response in Glioma Patients. World Neurosurg. (2019) 132:e455–62. 10.1016/j.wneu.2019.08.117 31470166

[60] Werner JM Kuhl S Ulrich K Krischek B Stavrinou P Goldbrunner R. Expression of CD40 Correlates Negatively with Overall and Progression-Free Survival of Low- and High-Grade Gliomas. World Neurosurg. (2019) 130:e17–25. 10.1016/j.wneu.2019.05.112 31125770

[61] Long S Li M Liu J Yang Y Li G. Identification of immunologic subtype and prognosis of GBM based on TNFSF14 and immune checkpoint gene expression profiling. Aging. (2020) 12:7112–28. 10.18632/aging.103065 32310827 PMC7202515

[62] Kong T Ahn R Yang K Zhu X Fu Z Morin G. CD44 Promotes PD-L1 Expression and Its Tumor-Intrinsic Function in Breast and Lung Cancers. Cancer Res. (2020) 80:444–57. 10.1158/0008-5472.CAN-19-1108 31722999

[63] Lee Y Shin JH Longmire M Wang H Kohrt HE Chang HY. CD44+ Cells in head and neck squamous cell carcinoma suppress T-cell-mediated immunity by selective constitutive and inducible expression of PD-L1. Clin Cancer Res. (2016) 22:3571–81. 10.1158/1078-0432.CCR-15-2665 26864211 PMC5623594

[64] Masugi Y Nishihara R Yang J Mima K da Silva A Shi Y. Tumour CD274 (PD-L1) expression and T cells in colorectal cancer. Gut. (2017) 66:1463–73. 10.1136/gutjnl-2016-311421 27196573 PMC5097696

[65] Stephen TL Payne KK Chaurio RA Allegrezza MJ Zhu H Perez-Sanz J. SATB1 Expression Governs Epigenetic Repression of PD-1 in Tumor-Reactive T Cells. Immunity. (2017) 46:51–64. 10.1016/j.immuni.2016.12.015 28099864 PMC5336605

[66] Wu C Thalhamer T Franca RF Xiao S Wang C Hotta C. Galectin-9-CD44 interaction enhances stability and function of adaptive regulatory T cells. Immunity. (2014) 41:270–82. 10.1016/j.immuni.2014.06.011 25065622 PMC4219323

[67] Bollyky PL Falk BA Long SA Preisinger A Braun KR Wu RP. CD44 costimulation promotes FoxP3+ regulatory T cell persistence and function *via* production of IL-2, IL-10, and TGF-beta. J Immunol. (2009) 183:2232–41. 10.4049/jimmunol.0900191 19635906 PMC3057032

[68] Trejdosiewicz LK Morton R Yang Y Banks RE Selby PJ Southgate J. Interleukins 4 and 13 upregulate expression of cd44 in human colonic epithelial cell lines. Cytokine. (1998) 10:756–65. 10.1006/cyto.1998.0361 9811528

[69] Ibrahim EM Stewart RL Corke K Blackett AD Tidy JA Wells M. Upregulation of CD44 expression by interleukins 1, 4, and 13, transforming growth factor-beta1, estrogen, and progestogen in human cervical adenocarcinoma cell lines. Int J Gynecol Cancer. (2006) 16:1631–42. 10.1111/j.1525-1438.2006.00637.x 16884377

