# Supplementary figures and images for: CD44-Mediated Poor Prognosis in Glioma Is Associated With M2-Polarization of Tumor-Associated Macrophages and Immunosuppression
Source: Front Surg. 2022 Feb 3;8:775194. doi: 10.3389/fsurg.2021.775194 (PMC8850306; doi:10.3389/fsurg.2021.775194)

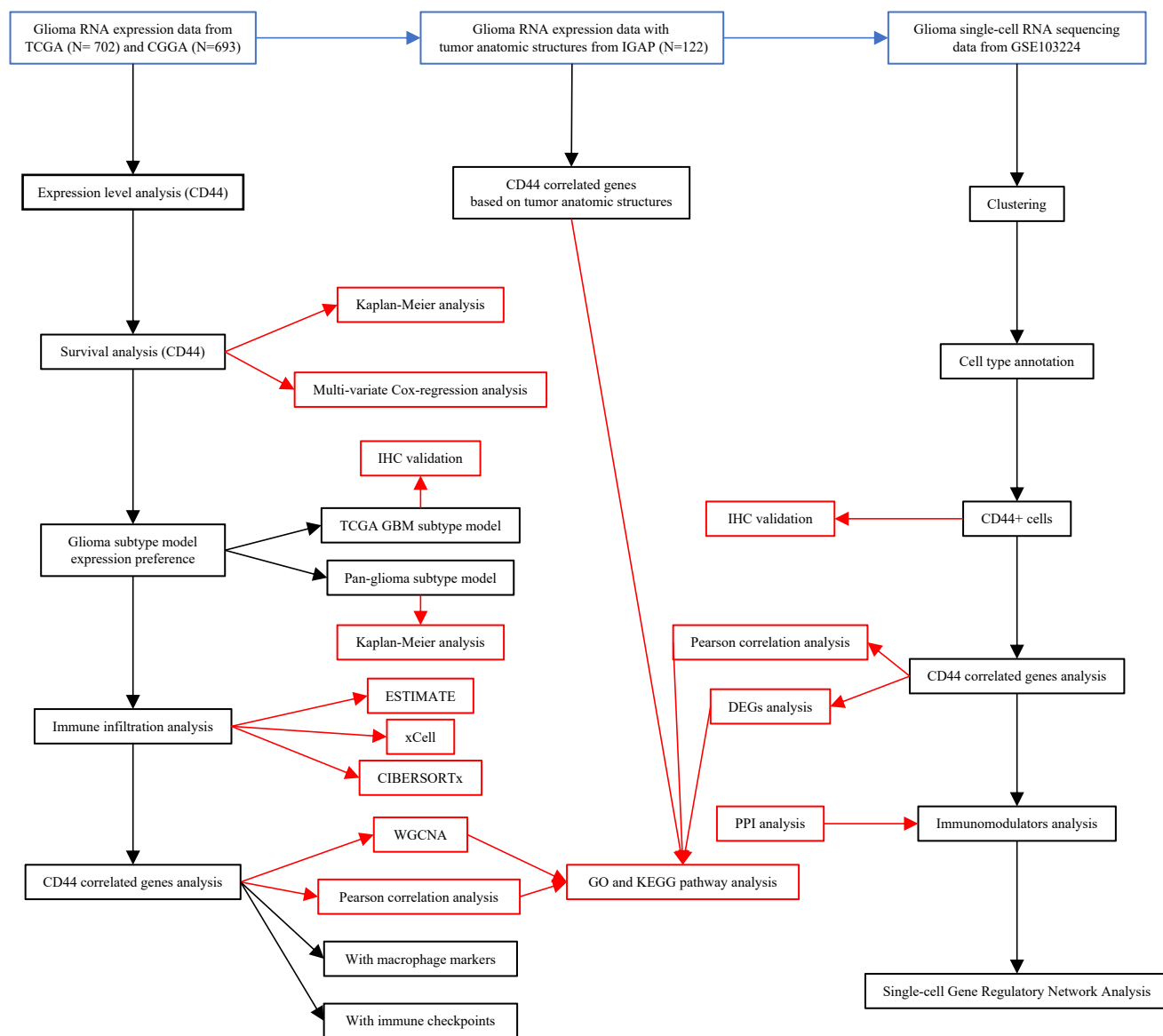

Supplement: Supplementary file 1 [file Data_Sheet_1.PDF]

Normal cerebral cortex  
Patient id: 2523

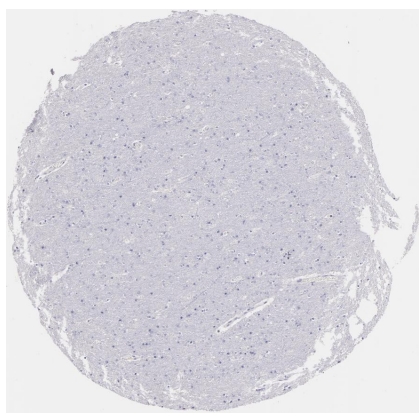

Low grade glioma  
Patient id: 34

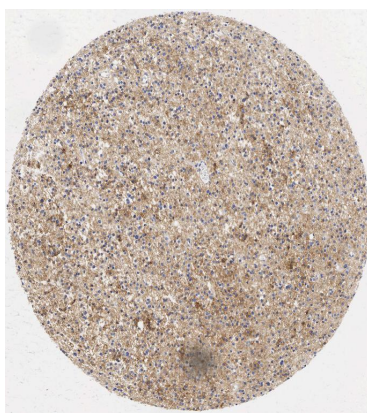

High grade glioma  
Patient id: 3091

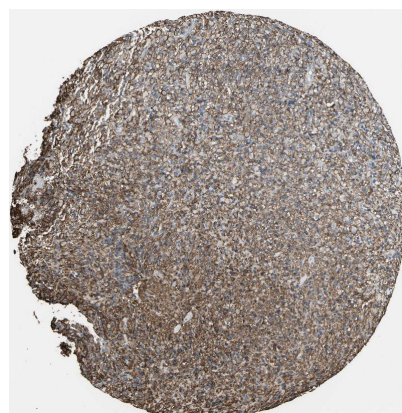

Supplement: Supplementary file 2 [file Data_Sheet_2.PDF]

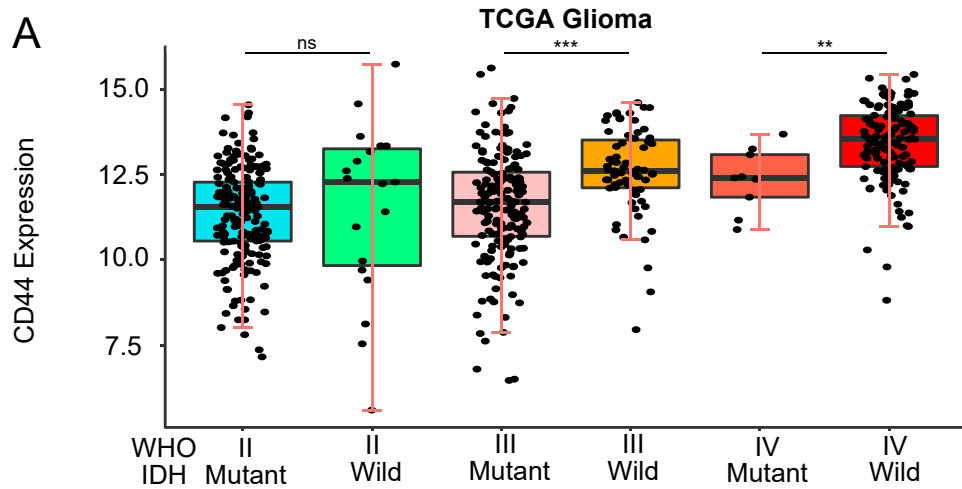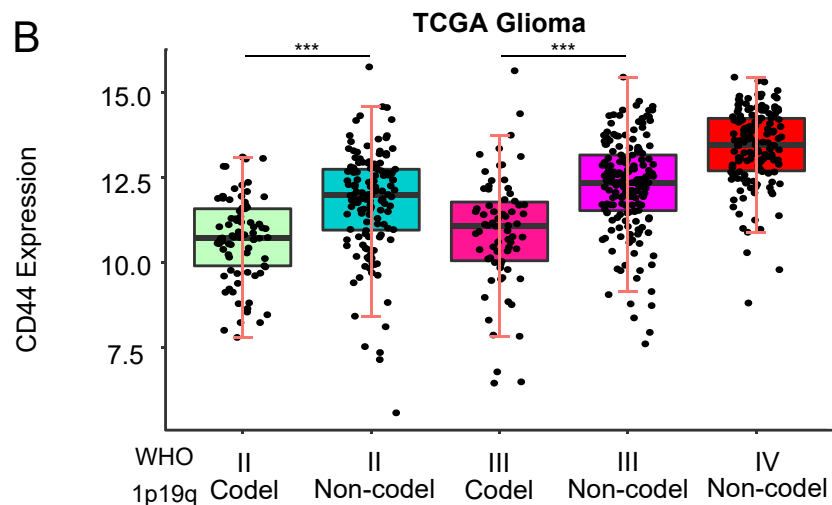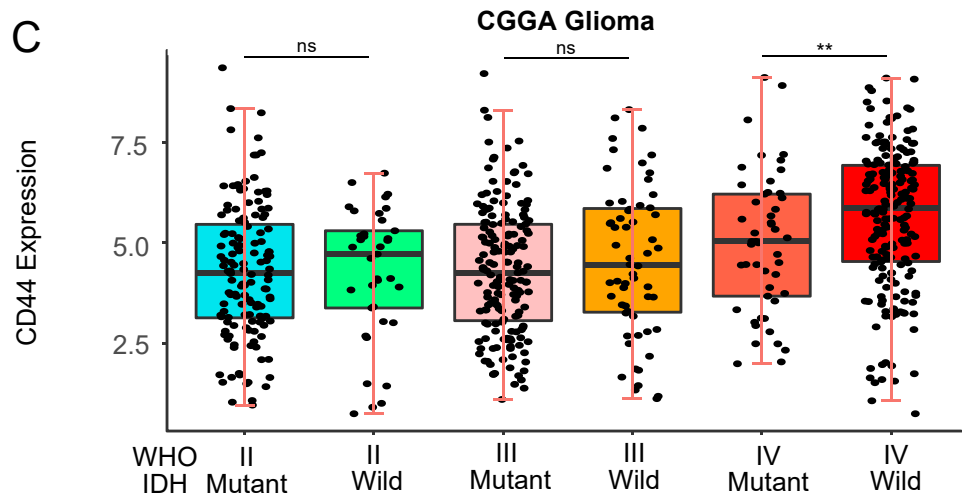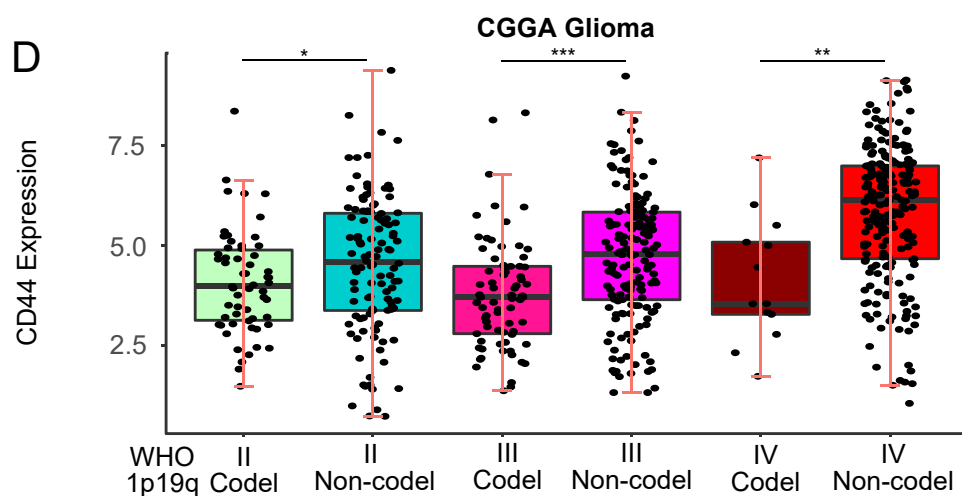

Supplement: Supplementary file 3 [file Data_Sheet_3.PDF]

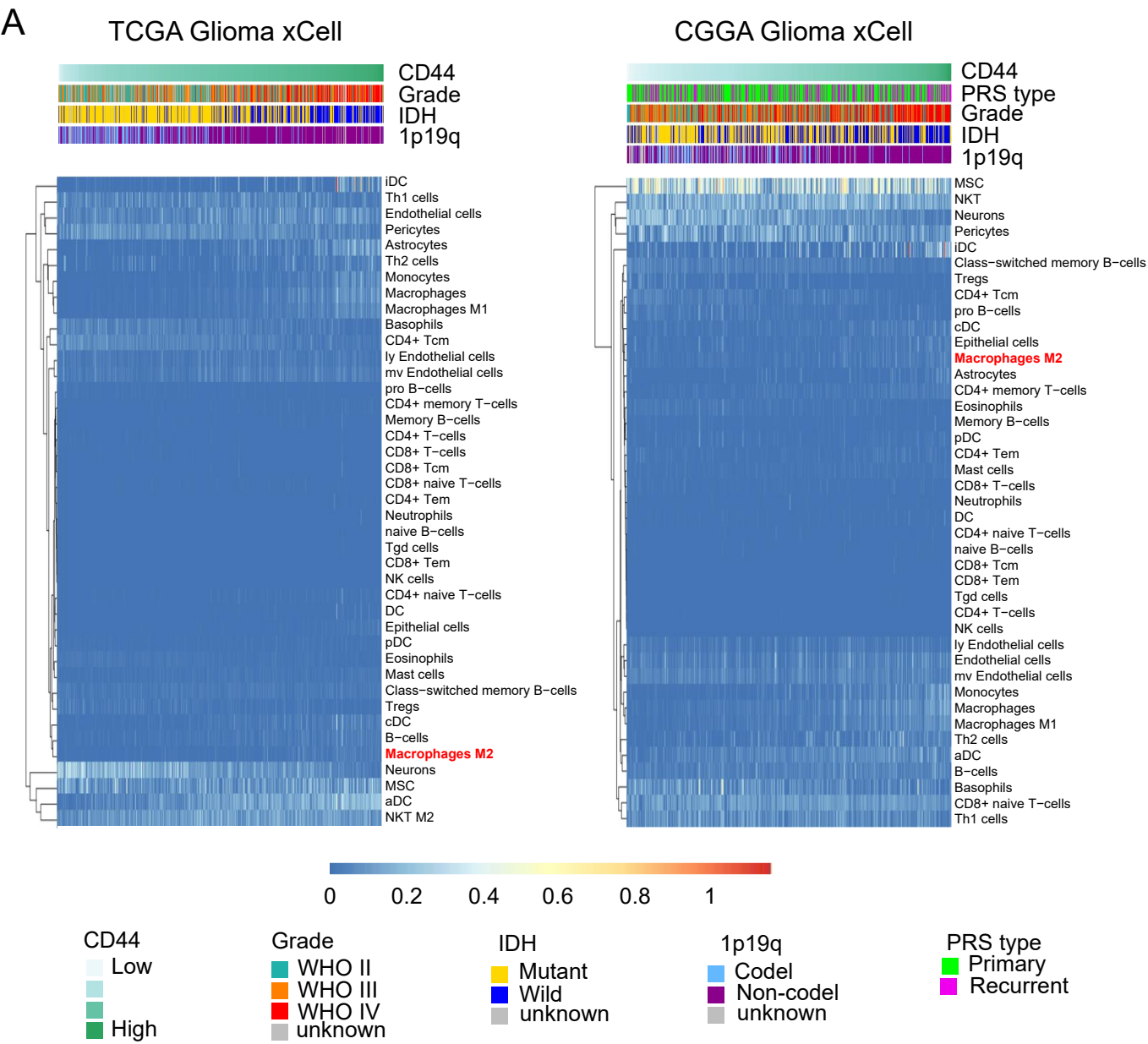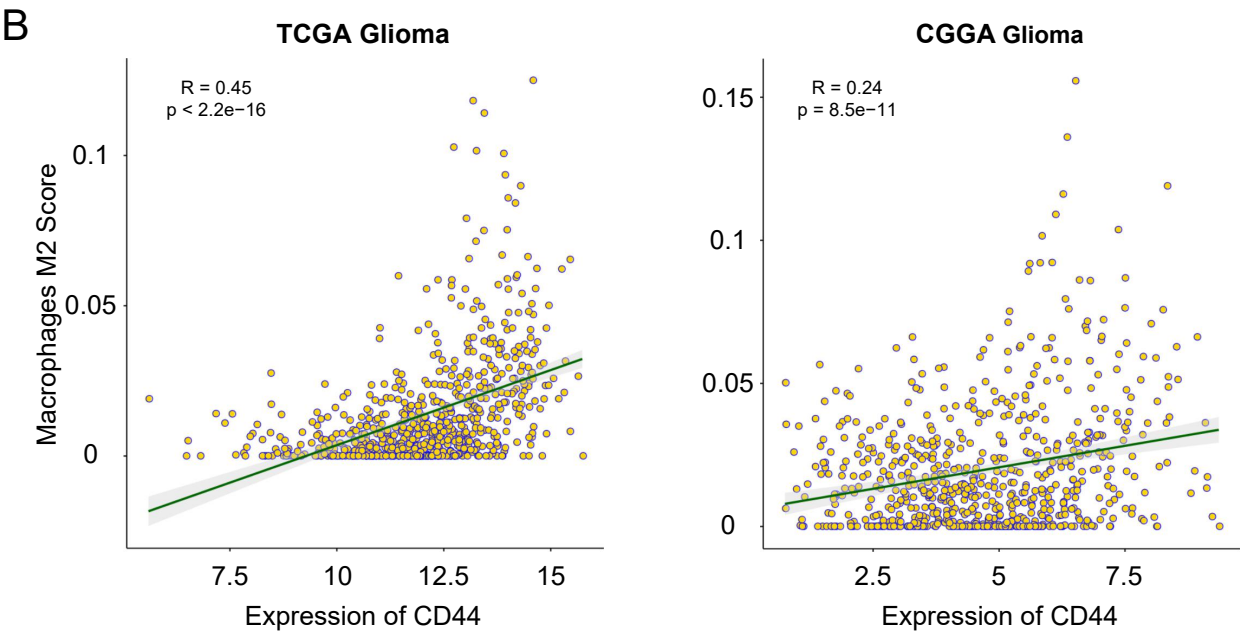

Supplement: Supplementary file 4 [file Data_Sheet_4.PDF]

A

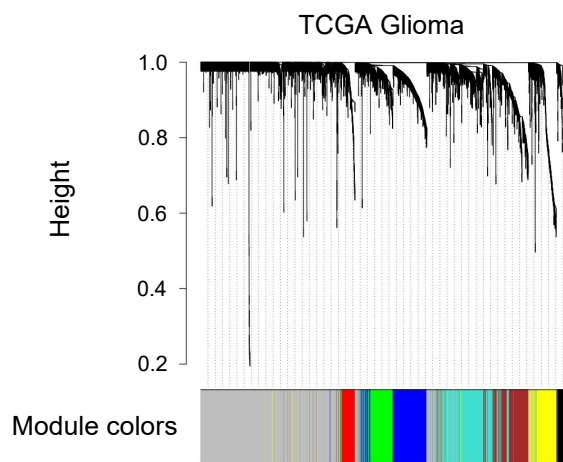

B

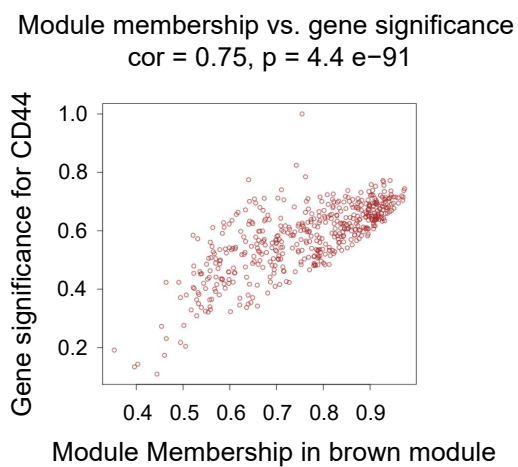

C

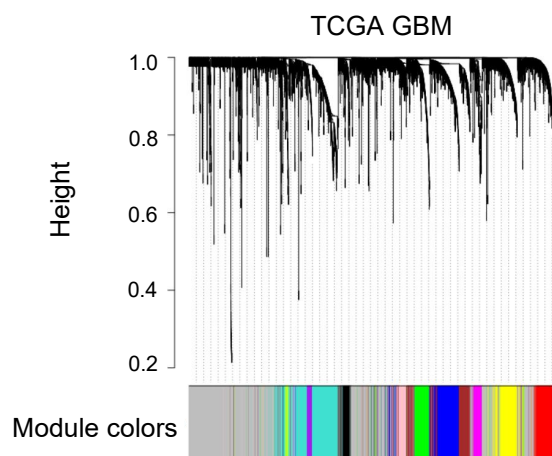

D

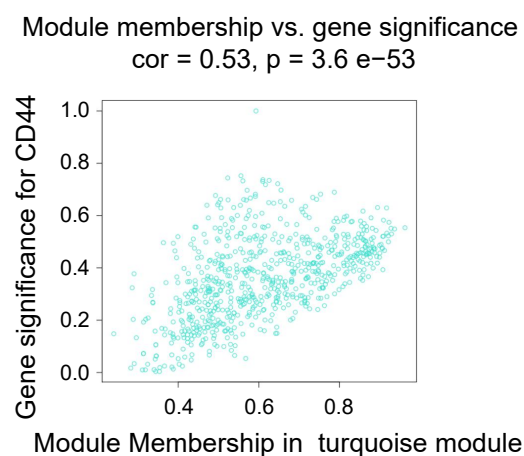

E

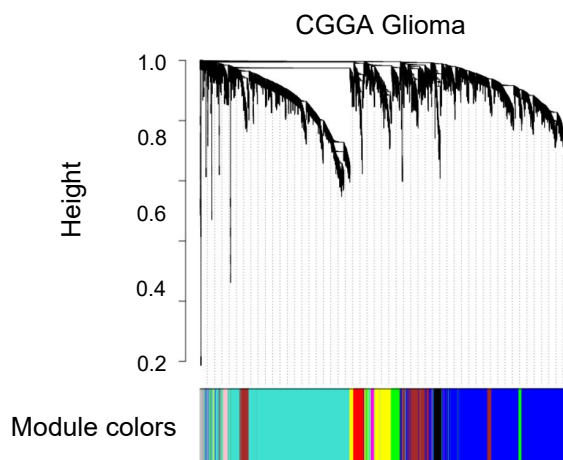

F

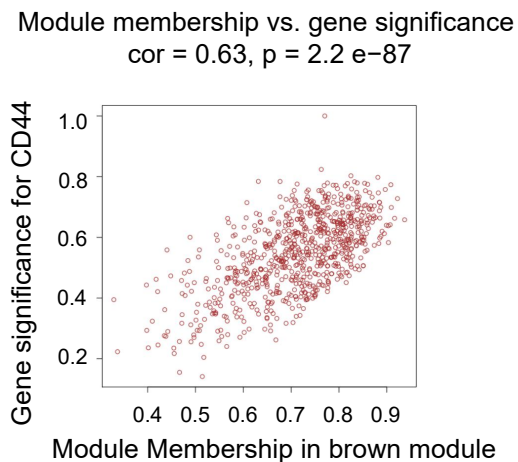

G

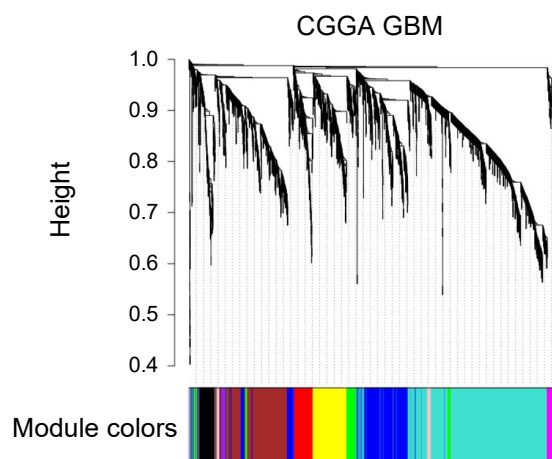

H

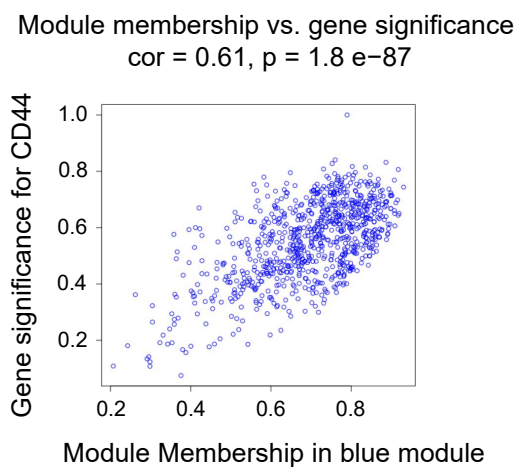

Supplement: Supplementary file 5 [file Data_Sheet_5.PDF]

A

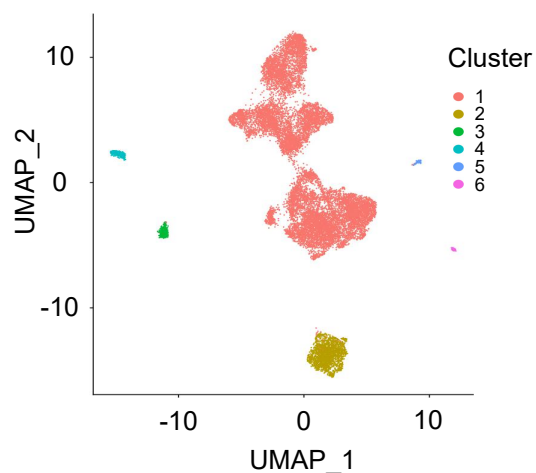

B

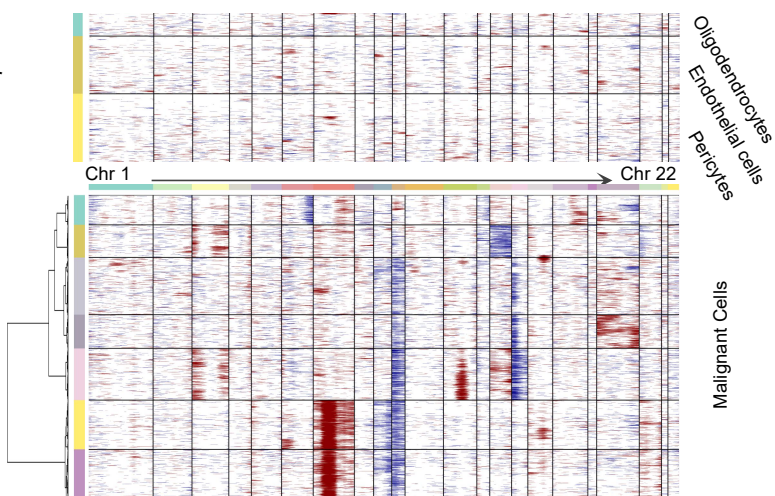

C

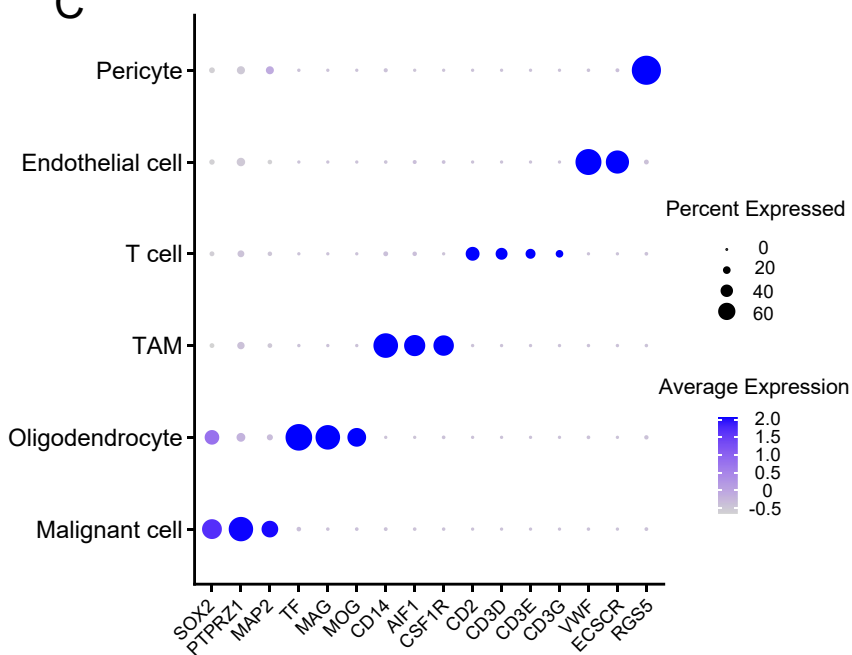

D

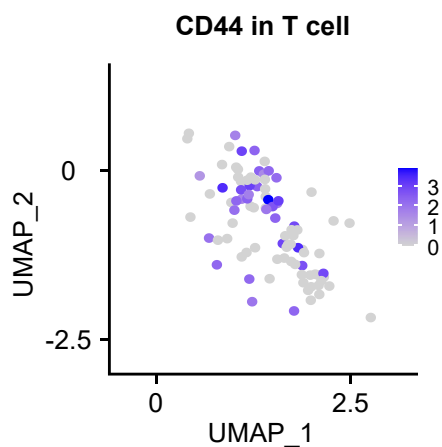

E

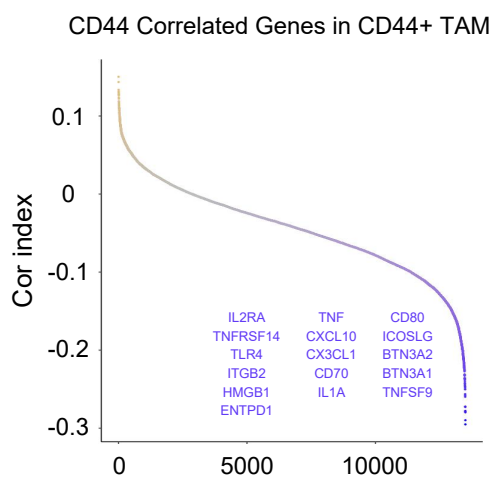

F

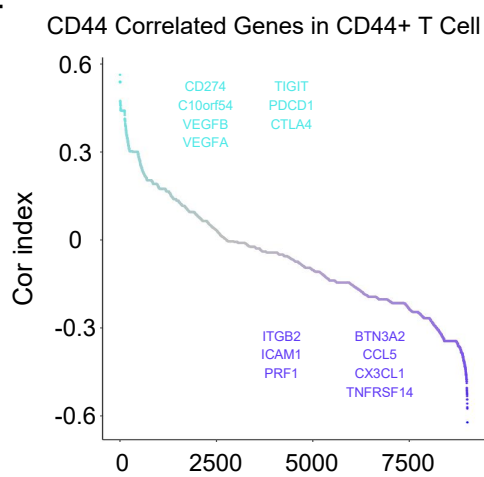

Supplement: Supplementary file 7 [file Data_Sheet_7.PDF]

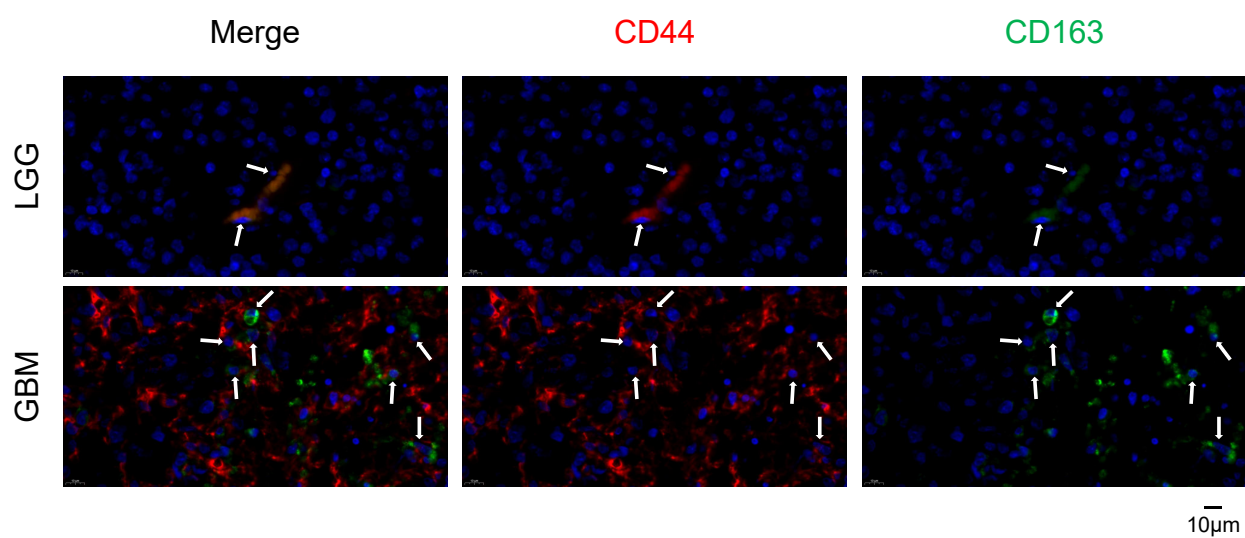

Supplement: Supplementary file 8 [file Data_Sheet_8.PDF]

A

TCGA Glioma

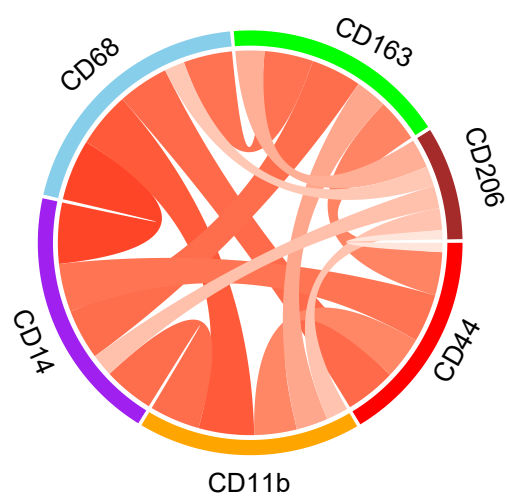

B

TCGA GBM

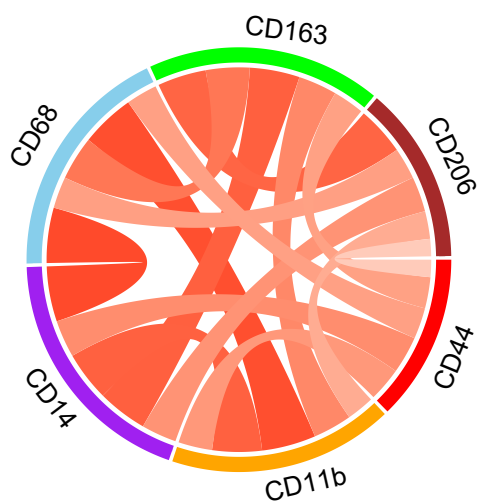

C

CGGA Glioma

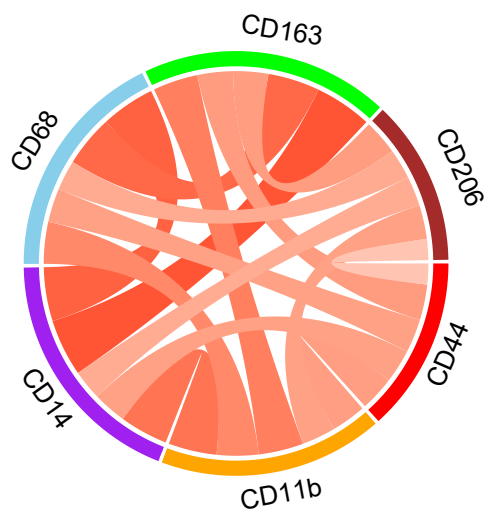

D

CGGA GBM

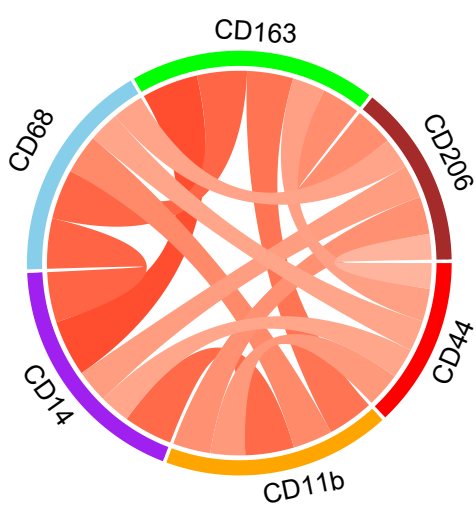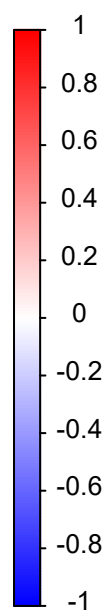

Supplement: Supplementary file 9 [file Data_Sheet_9.PDF]

A

TCGA Glioma

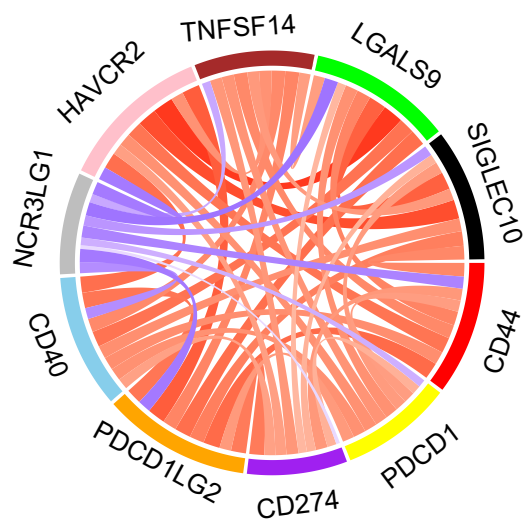

B

TCGA GBM

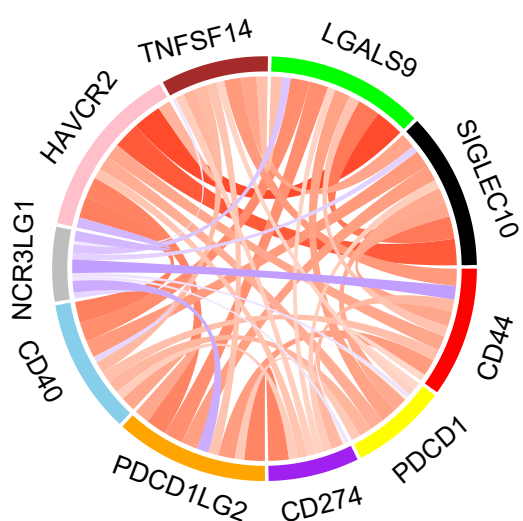

C

CGGA Glioma

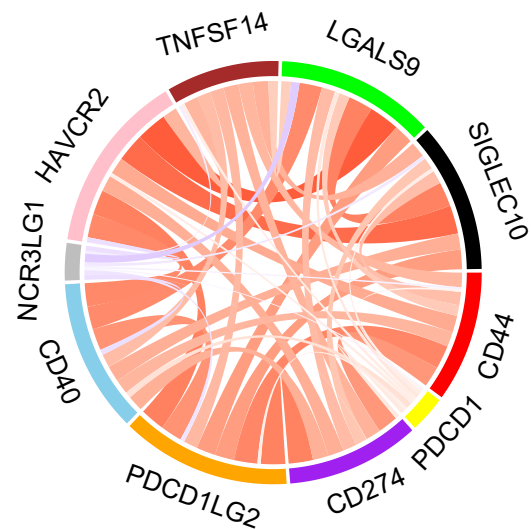

D

CGGA GBM

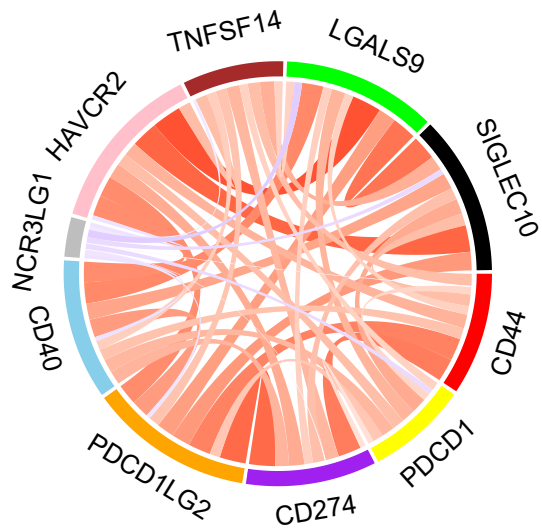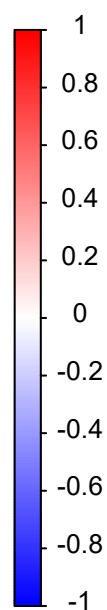

Supplement: Supplementary file 10 [file Data_Sheet_10.PDF]

A

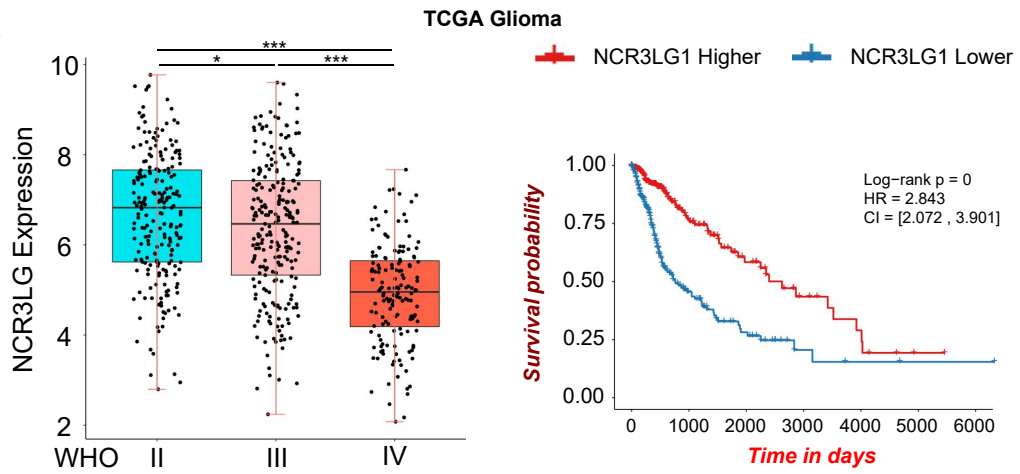

B

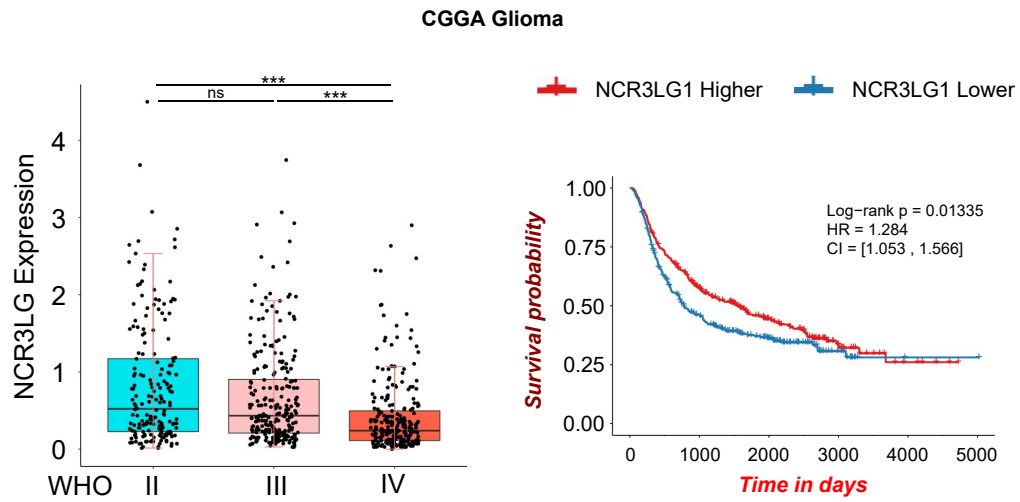

Supplement: Supplementary file 11 [file Data_Sheet_11.PDF]

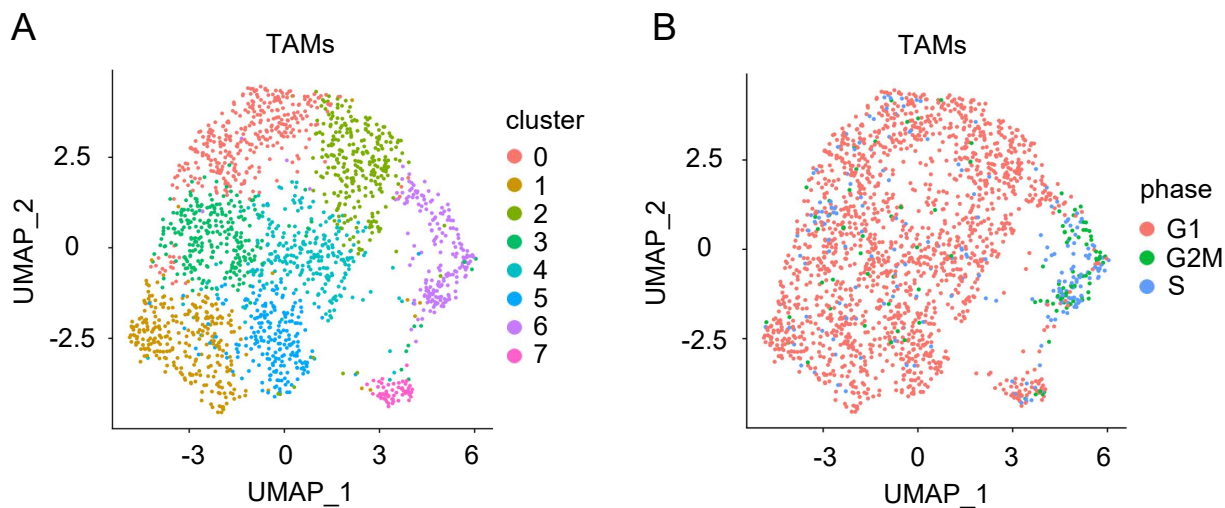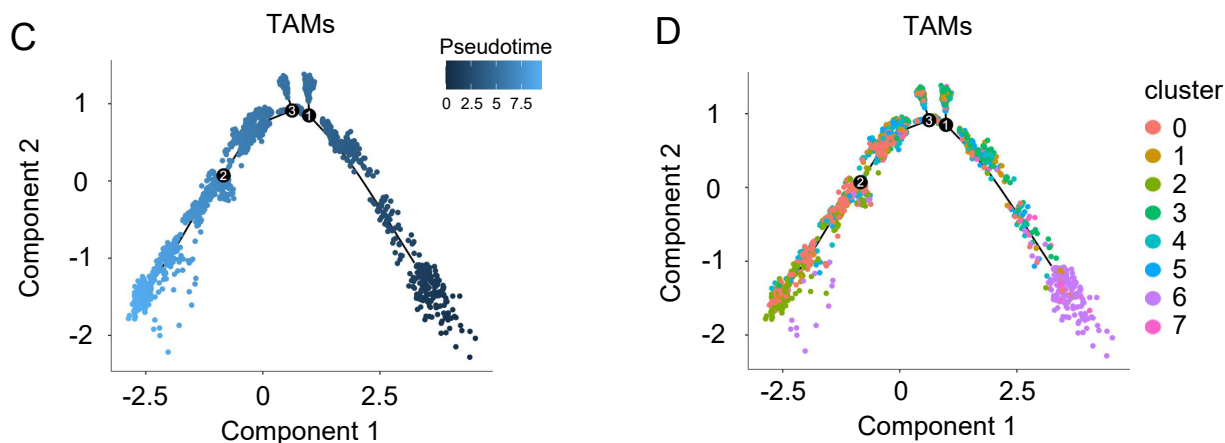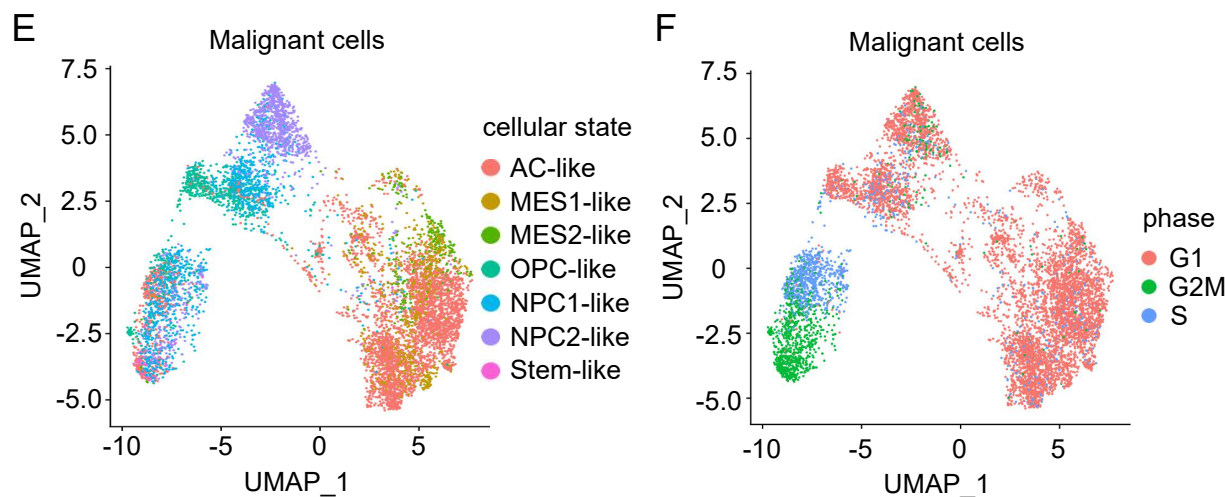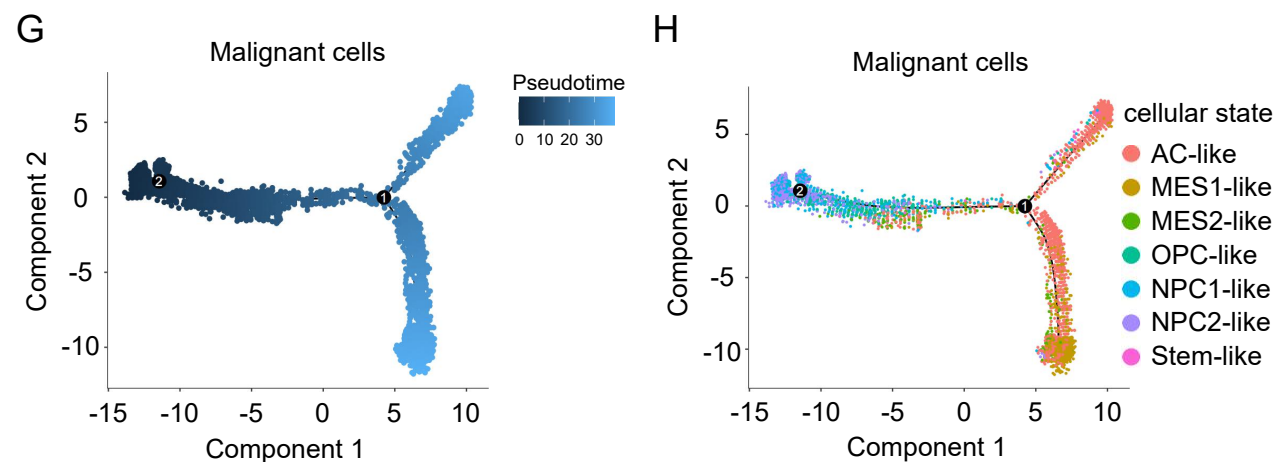

Supplement: Supplementary file 12 [file Data_Sheet_12.PDF]
