# Supplementary material for: CD44-Mediated Poor Prognosis in Glioma Is Associated With M2-Polarization of Tumor-Associated Macrophages and Immunosuppression
Source: Front Surg. 2022 Feb 3;8:775194. doi: 10.3389/fsurg.2021.775194 (PMC8850306; doi:10.3389/fsurg.2021.775194)
Supplement: Supplementary file 6 [file Data_Sheet_6.PDF]

A

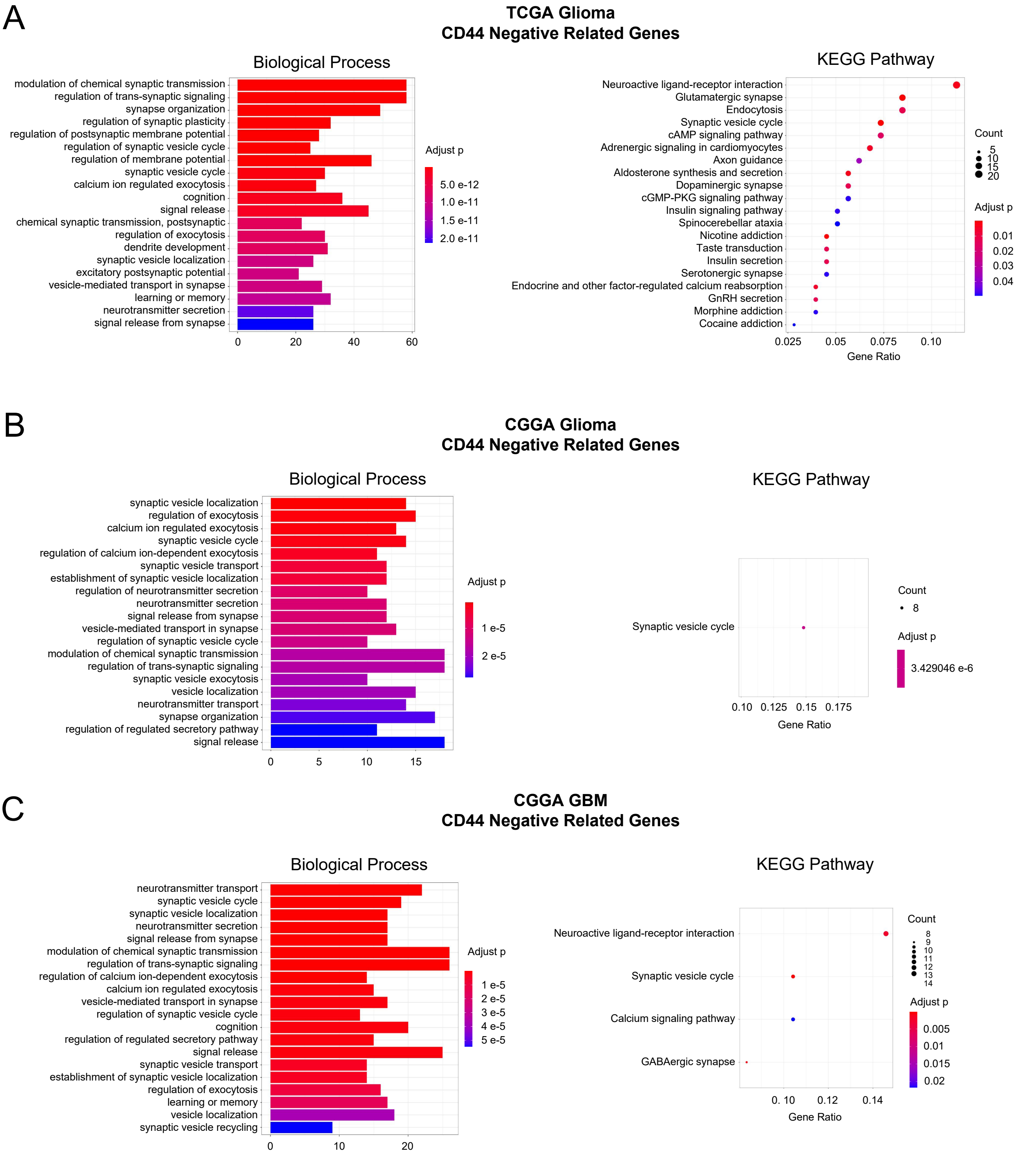
TCGA Glioma
CD44 Negative Related Genes
Biological Process
modulation of chemical synaptic transmission
regulation of trans-synaptic signaling
synapse organization
regulation of synaptic plasticity
regulation of postsynaptic membrane potential
regulation of synaptic vesicle cycle
regulation of membrane potential
synaptic vesicle cycle
calcium ion regulated exocytosis
cognition
signal release
chemical synaptic transmission, postsynaptic
regulation of exocytosis
dendrite development
synaptic vesicle localization
excitatory postsynaptic potential
vesicle-mediated transport in synapse
learning or memory
neurotransmitter secretion
signal release from synapse
0
20
40
60
Adjust p
5.0 e-12
1.0 e-11
1.5 e-11
2.0 e-11
KEGG Pathway
Neuroactive ligand-receptor interaction
Glutamatergic synapse
Endocytosis
Synaptic vesicle cycle
cAMP signaling pathway
Adrenergic signaling in cardiomyocytes
Axon guidance
Aldosterone synthesis and secretion
Dopaminergic synapse
cGMP-PKG signaling pathway
Insulin signaling pathway
Spinocerebellar ataxia
Nicotine addiction
Taste transduction
Insulin secretion
Serotonergic synapse
Endocrine and other factor-regulated calcium reabsorption
GnRH secretion
Morphine addiction
Cocaine addiction
0.025
0.05
0.075
0.10
Gene Ratio
Count
5
10
15
20
Adjust p
0.01
0.02
0.03
0.04
B
CGGA Glioma
CD44 Negative Related Genes
Biological Process
synaptic vesicle localization
regulation of exocytosis
calcium ion regulated exocytosis
synaptic vesicle cycle
regulation of calcium ion-dependent exocytosis
synaptic vesicle transport
establishment of synaptic vesicle localization
regulation of neurotransmitter secretion
neurotransmitter secretion
signal release from synapse
vesicle-mediated transport in synapse
regulation of synaptic vesicle cycle
modulation of chemical synaptic transmission
regulation of trans-synaptic signaling
synaptic vesicle exocytosis
vesicle localization
neurotransmitter transport
synapse organization
regulation of regulated secretory pathway
signal release
0
5
10
15
Adjust p
1 e-5
2 e-5
KEGG Pathway
Synaptic vesicle cycle
0.10
0.125
0.15
0.175
Gene Ratio
Count
8
Adjust p
3.429046 e-6
C
CGGA GBM
CD44 Negative Related Genes
Biological Process
neurotransmitter transport
synaptic vesicle cycle
synaptic vesicle localization
neurotransmitter secretion
signal release from synapse
modulation of chemical synaptic transmission
regulation of trans-synaptic signaling
regulation of calcium ion-dependent exocytosis
calcium ion regulated exocytosis
vesicle-mediated transport in synapse
regulation of synaptic vesicle cycle
cognition
regulation of regulated secretory pathway
signal release
synaptic vesicle transport
establishment of synaptic vesicle localization
regulation of exocytosis
learning or memory
vesicle localization
synaptic vesicle recycling
0
10
20
Adjust p
1 e-5
2 e-5
3 e-5
4 e-5
5 e-5
KEGG Pathway
Neuroactive ligand-receptor interaction
Synaptic vesicle cycle
Calcium signaling pathway
GABAergic synapse
0. 10
0.12
0.14
Gene Ratio
Count
8
9
10
11
12
13
14
Adjust p
0.005
0.01
0.015
0.02
