# Supplementary material for: CD44-Mediated Poor Prognosis in Glioma Is Associated With M2-Polarization of Tumor-Associated Macrophages and Immunosuppression
Source: Front Surg. 2022 Feb 3;8:775194. doi: 10.3389/fsurg.2021.775194 (PMC8850306; doi:10.3389/fsurg.2021.775194)
Supplement: Supplementary file 13 [file Data_Sheet_13.docx]

***Supplementary Materials***

**Supplementary Figure 1 |** Flow diagram of this study.

**Supplementary Figure 2 |** The immunohistochemistry staining of normal brain and glioma tissues. The high-grade glioma sample has the highest *CD44* protein expression level, but the normal brain sample has the lowest *CD44* protein expression.

**Supplementary Figure 3 |** *CD44* is highly expressed in IDH wild and 1p19q non-codeletion glioma. *CD44* significantly increases in IDH wild glioma form TCGA (A) and CGGA (C). *CD44* significantly increases in 1p19q Non-codeletion glioma form TCGA (B) and CGGA (D). Tested by t-test: p < 0.001, ***; p < 0.01, **; p < 0.05, *; p ≥ 0.05, ns.

**Supplementary Figure 4 |** Correlation between *CD44* expression and glioma microenvironment. (A) Component of 41 cell types infiltrated into glioma were analyzed by xCell in TCGA and CGGA glioma. Macrophages M2 was labeled in red. (B) Correlation of *CD44* expression with Macrophages M2 score generated by xCell analysis in the TCGA and CGGA glioma. The CD44 expression level is positively correlated with M2 macrophage score in glioma.

**Supplementary Figure 5 |** WGCNA analysis of significant modules. WGCNA cluster dendrogram on all samples groups genes into distinct driver modules in TCGA-glioma (A), TCGA-GBM (C), CGGA-glioma (E) and CGGA-GBM (G). Co-expression distance (topology overlap) between genes (y-axis) and to genes (x-axis). Gene modules are color coded. Scatterplot of gene significance for *CD44* mRNA expression level (y-axis) vs. module membership in module of interest (x-axis) in TCGA-glioma (B), TCGA-GBM (D), CGGA-glioma (F) and CGGA-GBM (H).

**Supplementary Figure 6 |** Enrichment analysis of *CD44* negative related genes in TCGA Glioma (A), CGGA Glioma (B) and CGGA GBM (C).

**Supplementary Figure 7 |** Cellular atlas of GBM. (A) UMAP reduction of all cells from 7 GBM patients. (B) Copy number variations (CNVs) estimation. Compared to normal stromal cells, malignant tumor cells have significant CNVs. (C) Cell type annotation by known cell markers. (D) Expression of *CD44* in T cells is projected on UMAP reduction. *CD44* correlated genes in *CD44*+ TAMs (E). Blue colored genes are immune stimulator genes and their expression negatively relates with *CD44*. *CD44* correlated genes in *CD44*+ T cells (F). Cyan labeled genes are immunosuppressive genes, whose expression level positively correlates with *CD44*; blue colored genes are immune stimulator genes and their expression negatively relates with *CD44*.

**Supplementary Figure 8 |** The immunofluorescence staining of glioma samples. *CD44* is colored in red and *CD163* is colored in green. *CD44+CD163+* cells are pointed out by white arrows. Scale, 10 μm.

**Supplementary Figure 9 |** Relationship between *CD44* and markers of macrophages. Correlation of *CD44* and macrophage markers including *CD11b*, *CD14*, *CD68*, *CD163*, and *CD206* in TCGA-glioma (A), TCGA-GBM (B), CGGA-glioma (C) and CGGA-GBM (D). *CD44* expression level positively correlates with *CD11b*, *CD14*, *CD68*, *CD163*, and *CD206.*

**Supplementary Figure 10 |** Correlation of *CD44* and immune checkpoints. Relationship between *CD44* and immune checkpoints were shown in TCGA-glioma (A), TCGA-GBM (B), CGGA-glioma (C) and CGGA-GBM (D). *CD44* expression level is positively correlated with *CD40*, *CD274*, *PDCD1*, *PDCD1LG2*, *HAVCR2*, *TNFSF14*, *LGALS9* and *SIGLEC10*, but negatively correlated with *NCR3LG1*.

**Supplementary Figure 11 |** Role of *NCR3LG1* in glioma. *NCR3LG1* is negative related to WHO grade malignancy glioma and positive correlated with prognosis in glioma from TCGA (A) and CGGA (B). Tested by t-test: p < 0.001, ***; p < 0.01, **; p < 0.05, *; p ≥ 0.05, ns.

**Supplementary Figure 12 |** Trajectory analysis of TAMs and tumor cells in GBM. Cell clusters (A) and cell cycle stages (B) of TAMs are projected on UMAP reduction. Trajectory of TAMs colored by pseudotime (C) and cell clusters (D). Cellular states (E) and cell cycle stages (F) of malignant tumor cells are projected on UMAP reduction. Trajectory of tumor cells colored by pseudotime (G) and cellular states (H).

**Supplementary Table 1 |** Enrichment analysis of significant gene modules from WGCNA analysis in glioma.

**Supplementary Table 2 |** Enrichment analysis of *CD44* related genes in TCGA glioma.

**Supplementary Table 3 |** Enrichment analysis of *CD44* related genes in CGGA glioma.

**Supplementary Table 4 |** Enrichment analysis of *CD44* related genes in IVY GBM.

**Supplementary Table 5 |** Enrichment analysis of *CD44* related genes in TAM.

**Supplementary Table 6 |** Enrichment analysis of *CD44* related genes in T cell.

**Supplementary Table 7 |** Enrichment analysis of *CD44* related genes in malignant cell.

**Supplementary Table 8 |** Correlated genes with *CD44* in single-cell RNA sequence data.

**Supplementary Table 9 |** Differentially expressed genes in non-responder vs. responder recurrent GBM treating with anti-*PD-1* immunotherapy.
